# Tet enzymes are essential for early embryogenesis and completion of embryonic genome activation

**DOI:** 10.15252/embr.202153968

**Published:** 2021-12-06

**Authors:** Julia Arand, H Rosaria Chiang, David Martin, Michael P Snyder, Julien Sage, Renee A Reijo Pera, Mark Wossidlo

**Affiliations:** ^1^ Center of Anatomy and Cell Biology Department of Cell and Developmental Biology Medical University of Vienna Vienna Austria; ^2^ Institute for Stem Cell Biology and Regenerative Medicine Stanford University Stanford CA USA; ^3^ Department of Pediatrics Stanford University Stanford CA USA; ^4^ Department of Genetics Stanford University Stanford CA USA; ^5^ Department of Obstetrics & Gynecology Stanford University Stanford CA USA; ^6^ Present address: McLaughlin Research Institute Great Falls MT USA

**Keywords:** DNA methylation reprogramming, embryonic genome activation, Tet enzymes, totipotency, transposable elements, Chromatin, Transcription & Genomics, Development, Post-translational Modifications & Proteolysis

## Abstract

Mammalian development begins in transcriptional silence followed by a period of widespread activation of thousands of genes. DNA methylation reprogramming is integral to embryogenesis and linked to Tet enzymes, but their function in early development is not well understood. Here, we generate combined deficiencies of all three Tet enzymes in mouse oocytes using a morpholino‐guided knockdown approach and study the impact of acute Tet enzyme deficiencies on preimplantation development. Tet1–3 deficient embryos arrest at the 2‐cell stage with the most severe phenotype linked to Tet2. Individual Tet enzymes display non‐redundant roles in the consecutive oxidation of 5‐methylcytosine to 5‐carboxylcytosine. Gene expression analysis uncovers that Tet enzymes are required for completion of embryonic genome activation (EGA) and fine‐tuned expression of transposable elements and chimeric transcripts. Whole‐genome bisulfite sequencing reveals minor changes of global DNA methylation in Tet‐deficient 2‐cell embryos, suggesting an important role of non‐catalytic functions of Tet enzymes in early embryogenesis. Our results demonstrate that Tet enzymes are key components of the clock that regulates the timing and extent of EGA in mammalian embryos.

## Graphical abstract

A morpholino‐based knockdown approach reveals an essential function of Tet enzymes in mouse preimplantation development and embryonic genome activation.

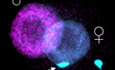

## Introduction

Mammalian life starts in transcriptional silence with an oocyte‐to‐embryo transition that encompasses the fusion of the egg and sperm, migration and fusion of the germ cell pronuclei, and a series of cleavage divisions culminating in the activation of the unique embryonic genome followed by compaction of blastomeres to form a morula and differentiation of the first cell lineages—the trophectoderm and inner cell mass (Rossant & Tam, 2009). Embryo development is remarkable in that the oocyte must provide all required resources to carry out the complex developmental pathways in the absence of transcription prior to embryonic genome activation (EGA) (Braude *et al*, 1988; Zhang & Smith, 2015).

DNA methylation reprogramming of oocyte and sperm genomes during early mammalian embryogenesis ensures the development of totipotent and pluripotent preimplantation embryos capable of contributing to diverse cell lineages (Messerschmidt *et al*, 2014; Eckersley‐Maslin *et al*, 2018). Shortly after fertilization, a well‐orchestrated and yet not fully understood combination of active and passive DNA demethylation, as well as *de novo* methylation reprograms the DNA methylation landscape of paternal and maternal genomes in the zygote (Ladstatter & Tachibana, 2019). Passive DNA demethylation during replication through non‐maintenance of 5‐methylcytosine (5mC) has been shown to have the biggest impact on global DNA methylation levels in preimplantation development (Guo *et al*, 2014; Shen *et al*, 2014) and is accompanied by selective maintenance and *de novo* methylation (Arand *et al*, 2015; Amouroux *et al*, 2016). Conversely, active DNA demethylation by enzymatic modification/removal of 5mC was reported to play an important role in the transcriptional regulation of specific genomic loci, but a rather moderate role in global DNA methylation reprogramming, and can involve ten‐eleven‐translocation (Tet) enzymes (Peat *et al*, 2014; Shen *et al*, 2014). Moreover, Tet enzyme activity in the zygote has been shown to protect certain CpGs from methylation buildup by DNA *de novo* methyltransferases (Peat *et al*, 2014). Tet proteins catalyze the enzymatic oxidation of 5mC to 5‐hydroxymethylcytosine (5hmC), 5‐formylcytosine (5fC), and 5‐carboxylcytosine (5caC) in a consecutive manner (He *et al*, 2011; Ito *et al*, 2011). In mammals, three Tet enzymes (Tet1, Tet2, and Tet3) are differentially expressed during development and play distinct roles in different cell types (Ito *et al*, 2010; Gu *et al*, 2011; Koh *et al*, 2011; Moran‐Crusio *et al*, 2011; Wossidlo *et al*, 2011; Moyon *et al*, 2021).

Tet3 is the highest expressed Tet protein in oocytes, and knockdown (KD) and knockout (KO) studies in mouse zygotes have shown that Tet3 mediates the conversion of 5mC to 5hmC (Gu *et al*, 2011; Wossidlo *et al*, 2011; Yu *et al*, 2013). Next to Tet3, Tet1 and Tet2 are also expressed during early preimplantation development (Wossidlo *et al*, 2011). Nevertheless, the importance of Tet enzymes and the oxidation of 5mC for preimplantation development is not clear, since so far, no severe phenotype in preimplantation development for different Tet‐KO mouse models was reported. Observed Tet‐KO phenotypes are obvious during postimplantation development, with preimplantation embryos showing mild transcriptomic changes or developmental impairment in different contexts. Here, heterozygous Tet3‐KO mice displayed neonatal abnormalities, and Tet1^−/−^ and Tet2^−/−^ single KO embryos developed with postnatal malignancies (Dawlaty *et al*, 2011; Gu *et al*, 2011; Ko *et al*, 2011; Li *et al*, 2011). Tet1 + 3 double KO mice are characterized by abnormal early postimplantation phenotypes with variable gene expression and reduced developmental success during preimplantation development (Kang *et al*, 2015), and Tet1 + 2 double KO mice are characterized by smaller ovaries and strongly reduced fertility rates (Dawlaty *et al*, 2013). Furthermore, a conditional germline knockout of all three Tet1–3 enzymes (Tet‐TKO) developed beyond the implantation stage, despite altered expression of a few hundred genes at the blastocyst stage (Dai *et al*, 2016). Derived Tet‐TKO embryos showed severe gastrulation phenotypes and did not develop to term (Dai *et al*, 2016). These data so far point to an important role for Tet enzymes in later stages of development but not during the striking DNA reprogramming phase in early preimplantation embryos. Notably, the experimental design of the reported Tet‐KO studies involved conditional knockout strategies targeting the growth phase of gametes during spermatogenesis and oogenesis. Loss of Tet enzymes in maturing gametes might already impact their epigenome, with the potential to influence and alter embryonic development. Moreover, the Tet‐TKO and most other knockout studies were designed to create Tet‐null mutants lacking the catalytic activities of Tet enzymes and are not complete genomic deletions of these enzymes. In this context, it has been shown that gene modifications, which aim to create catalytically dead enzymes by truncation of catalytical domains, like the conditional truncations of Tet enzymes in the growing oocyte (Gu *et al*, 2011; Dai *et al*, 2016), can trigger the expression of related genes, which can severely obscure observed phenotypes (El‐Brolosy *et al*, 2019; Ma *et al*, 2019; Wilkinson, 2019). Interestingly, a study analyzing Tet‐TKO mESCs revealed an important role of Tet enzymes in regulating the transition into the totipotent 2‐cell embryo‐like state in mESCs (Lu *et al*, 2014) suggesting an active role of Tet enzymes in the regulation of totipotent cells.

Despite many years of extensive research, it still remained unclear why mammals would invest in a potentially very dangerous mechanism of active DNA demethylation that could cause severe problems in the newly developing embryo. In this study, we specifically investigated the role of Tet enzymes in the oocyte‐to‐embryo transition, by performing knockdown experiments in fully grown and genetically normal oocytes to determine the biological significance of Tet‐mediated DNA methylation reprogramming in mammalian preimplantation development. We also addressed the open question of whether Tet enzymes have overlapping roles in the enzymatic oxidation of 5mC and therefore can compensate each other interchangeably in the mammalian oocyte‐to‐embryo transition.

## Results and Discussion

### Tet1–3‐deficient mouse embryos arrest at the 2‐cell stage

**Figure 1:**
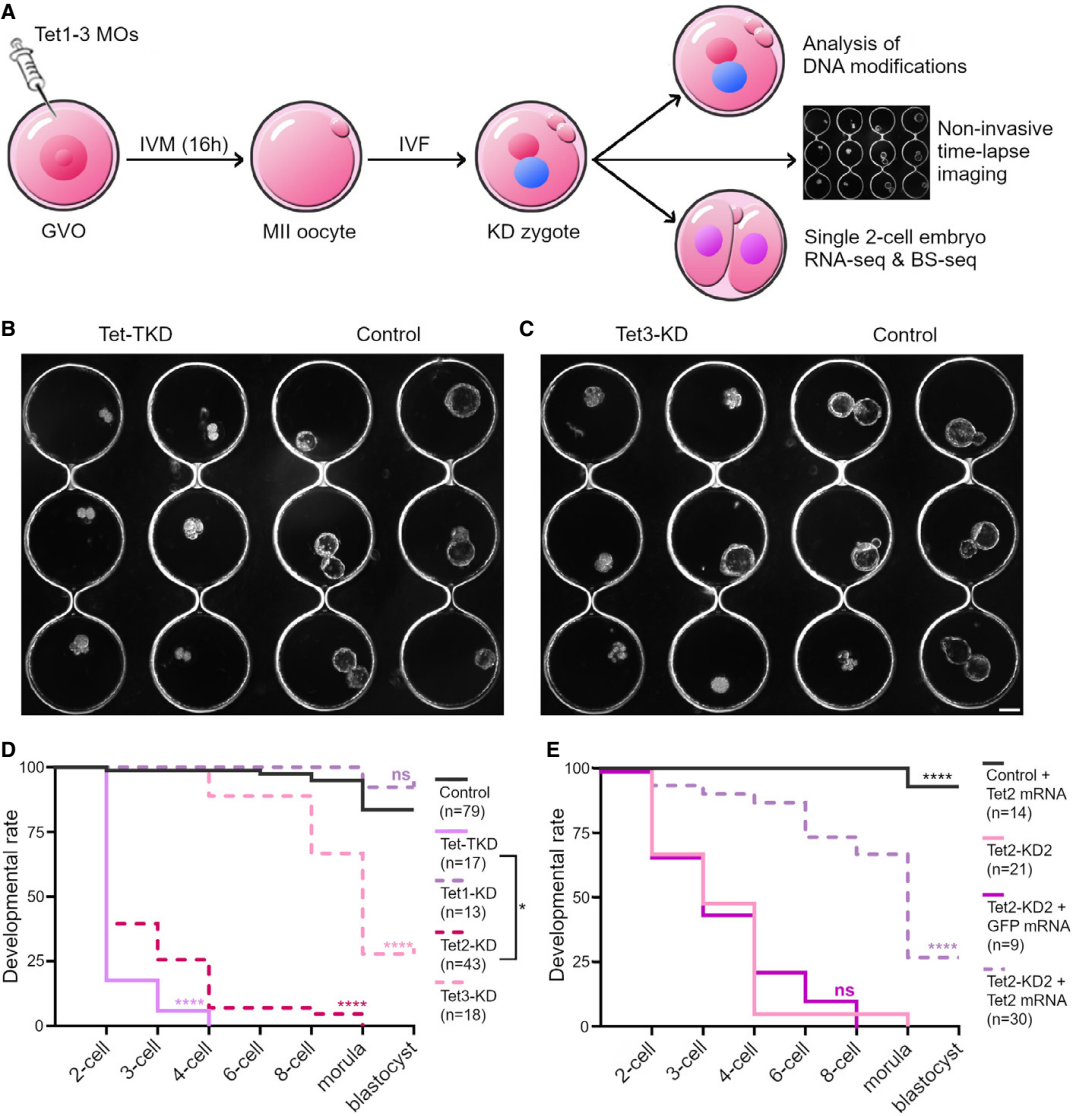
Tet enzyme‐deficient mouse embryos arrest primarily at the 2‐cell stage AExperimental setup: Mouse germinal vesicle oocytes (GVOs) were isolated and injected with Morpholinos (MOs) designed to target Tet1–3 mRNAs. After *in vitro* maturation (IVM), MII oocytes were fertilized by *in vitro* fertilization (IVF). Zygotes derived from knockdown (KD) and control‐MO injected oocytes were analyzed by (i) non‐invasive time‐lapse imaging to monitor the developmental potential of KD embryos, (ii) immunofluorescence to analyze changes in DNA methylation reprogramming and (iii) by single 2‐cell embryo RNA‐Seq & BS‐Seq to study the impact of Tet enzymes on embryonic gene activation (EGA) and DNA methylation reprogramming in the totipotent 2‐cell embryo.B, CRepresentative time‐lapse images of (B) Tet1–3 triple KD (Tet‐TKD), (C) Tet3‐KD, and control embryos at 3.5 days post‐fertilization. Scale bar = 100 µm.D, EDevelopmental rates of (D) control (control‐MO injected), Tet1‐, Tet2‐ and Tet3‐single KD and Tet‐TKD embryos and (E) control (control‐MO + Tet2 mRNA co‐injected), Tet2‐KD2 (Tet2 MO2 injected), Tet2‐KD2 + GFP‐mRNA and Tet2‐KD2 + Tet2 mRNA embryos until blastocyst stage. Indicated *P*‐values were calculated using log rank (Mantel‐Cox) test against (D) control embryos or (E) Tet2‐KD2 embryos or as indicated (ns = non‐significant, *P < 0.05, ****P < 0.0001; numbers of analyzed embryos for each condition are indicated in parentheses).

**Figure EV1:**
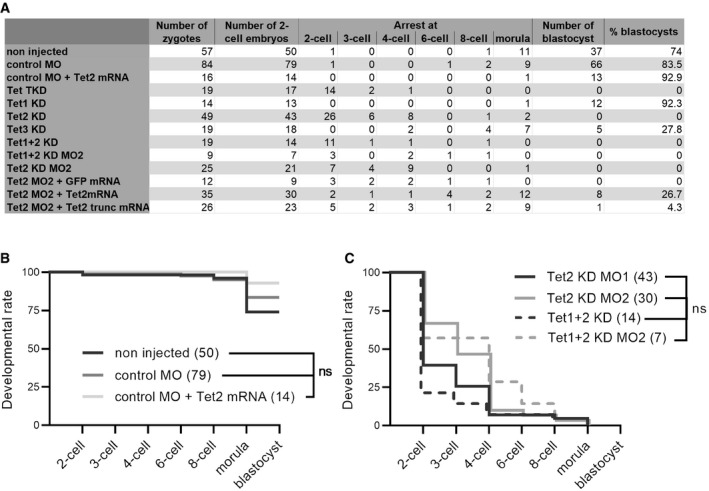
Developmental rates of embryos derived from microinjected oocytes ASummary of all analyzed samples (% blastocysts = % 2‐cell embryos developed to the blastocyst stage).B, CDevelopmental curves of selected sets of experimental groups starting from 2‐cell stage. (B) Comparison of control groups. (C) Comparison of Tet2‐MO1 and Tet2‐MO2 groups and Tet1 + 2 combined KDs. Indicated significances were tested using log rank (Mantel‐Cox) test (ns = non‐significant, numbers of analyzed embryos per experimental group are indicated in parenthesis).

In mouse oocytes and zygotes, Tet3 is highly expressed and Tet1 and Tet2 transcripts are present at low levels (Wossidlo *et al*, 2011). To analyze the developmental importance of Tet enzymes specifically in the oocyte‐to‐embryo transition, we designed knockdown experiments to deplete Tet enzymes in genetically normal mouse oocytes. Therefore, we microinjected different combinations of morpholinos (MOs) targeting Tet1–3 mRNAs into mouse germinal vesicle oocytes (GVOs) followed by *in vitro* maturation (IVM) and *in vitro* fertilization (IVF; Fig 1A, and Appendix Table S1). Subsequently, we performed non‐invasive time‐lapse imaging to follow the development of control‐MO‐injected and Tet1–3‐MO‐injected embryos until the blastocyst stage (Figs 1B–D and EV1A, and Movie EV1). Embryos derived from control‐MO injected oocytes developed into blastocysts with similar ratios as non‐injected embryos at expected mouse *in vitro* culture ratios (83.5%, see Figs 1B and C, and EV1A and B). Strikingly, the triple KD of all three Tet enzymes (Tet‐TKD) completely prevented blastocyst development (Fig 1B and D, see also Movie EV1). Moreover, the majority of Tet‐TKD embryos arrested at the 2‐cell stage (82%), and only a few advanced to the 4‐cell stage (Fig 1D).

Our observation using a morpholino‐based knockdown approach in fully grown oocytes stands in contrast to phenotypes observed upon germline deletion of all three Tet enzymes, where phenotypes are emerging in early postimplantation development (Dai *et al*, 2016). Both approaches have advantages and limitations and target different questions. The Tet‐TKO approach diminishes Tet proteins during the growth phase of the oocyte—a phase where massive DNA remethylation is still occurring and compensation mechanisms can intervene, while the morpholino‐based knockdown approach is effective in fully grown and transcriptionally silent oocytes, minimizing compensatory effects and only mimicking loss of Tet function specifically during the oocyte‐to‐embryo transition. Moreover, the Tet‐TKO and most single Tet‐KO models aimed to create genomic deletions of the catalytical domain of Tet enzymes leaving the possibility of the expression of a truncated product (Gu *et al*, 2011; Ko *et al*, 2011; Zhang *et al*, 2013; Dai *et al*, 2016), while the morpholino‐based KD diminishes the expression of the complete gene product. Albeit Cre/lox‐mediated KO approaches are highly specific and KD approaches bare possibilities of unspecific off‐target effects, KO strategies can lead to unspecific phenotypes due to non‐sense mediated decay of truncation constructs (El‐Brolosy *et al*, 2019; Ma *et al*, 2019; Wilkinson, 2019), whereas in MO‐based KD approaches, the full‐length mRNA is still generated but blocked for translation.

**Figure EV2:**
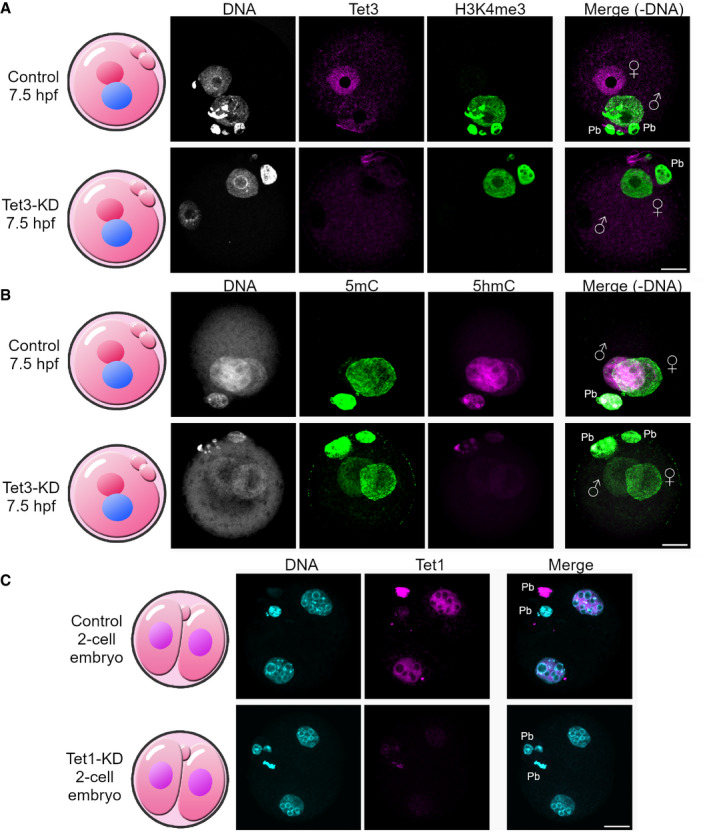
Analysis of Tet3‐ and Tet1‐knockdown efficiency A, BAnalysis of Tet3‐KD efficiency. (A) Representative images of Tet3 and H3K4me3 indirect immunofluorescence (IF) of 7.5 hpf zygotes derived from control or Tet3‐KD GVOs. Tet3 can be detected in the paternal pronucleus (the maternal pronucleus is marked by H3K4me3‐IF) of control derived zygotes, whereas Tet3 knockdown zygotes are negative for Tet3‐signal (*n* = 12). (B) Representative images of 5mC‐ and 5hmC‐IF of 7.5 hpf zygotes. The knockdown of Tet3 reduces the loss of 5mC and the gain of 5hmC in the paternal pronucleus (*n* = 14). Paternal and maternal pronuclei are indicated.CAnalysis of Tet1‐KD efficiency. Representative images of Tet1 IF of G2‐phase 2‐cell embryos (32 hpf) of control or Tet1‐KD‐derived GVOs (*n* = 12). Control embryos show nuclear Tet1 signal, whereas Tet1‐KD embryos show greatly reduced Tet1 signal. Data information: Paternal and maternal pronuclei are indicated, Pb = polar body, scale bar = 20 μm.

To decipher individual roles of Tet enzymes during early embryogenesis, we generated single Tet enzyme‐KD embryos. Tet3 is the most abundant Tet enzyme in mouse oocytes and mediates the conversion of 5mC to 5hmC (Gu *et al*, 2011; Wossidlo *et al*, 2011). Our Tet3 knockdown approach (Tet3‐KD) completely diminished Tet3 protein in the zygote and confirmed the importance of Tet3 for the 5mC to 5hmC conversion by drastically reducing 5hmC levels in early zygotes (Figs EV2A and B). Moreover, Tet3‐KD embryos demonstrated a decreased blastocyst formation (28% blastocysts, Fig 1C and D, and Movie EV2) suggesting that Tet3 is important for efficient preimplantation development, but not essential. Similar observations were made in Tet1 + 3 double KO mouse embryos (25% arrest before 8‐cell stage (Kang *et al*, 2015)) and Tet3‐KD bovine embryos (blastocyst rate decreases from 19 to 3% in Tet3‐KD (Cheng *et al*, 2019)). Regarding the only partially impaired preimplantation development of Tet3‐KD embryos, it is tempting to assume that passive DNA demethylation can compensate for Tet3 mediated demethylation. Here, while Tet3 might be targeted to specific genomic loci, this compensation process might be more unspecific, which can result in more variable expression profiles in early embryos (Kang *et al*, 2015) or, as observed in our experiments, in the early developmental arrest of few embryos. In line with that, hypermethylation in maternal Tet3‐KO embryos was reported to be largely diminished by the blastocyst stage (Inoue *et al*, 2015). The successful, although inefficient formation of Tet3‐KD blastocysts in contrast to the observed Tet‐TKD phenotype, which completely inhibits blastocyst formation, suggested that Tet1/2 also contribute significantly to preimplantation development. Therefore, we next performed single and double KD (DKD) experiments for Tet1 and Tet2. Tet1‐KD did not impact early embryogenesis, while Tet1 protein was successfully diminished at the 2‐cell stage (Figs 1D and EV2C). Remarkably, Tet2‐KD alone prevented normal preimplantation development to the blastocyst stage, with only a few embryos capable to develop to the morula stage (Fig 1D). The double KD of Tet1 and Tet2 (Tet1 + 2‐DKD) did not further elevate this phenotype (Fig EV1C) suggesting a negligible role of Tet1 in preimplantation development (see also (Dawlaty *et al*, 2011)).

These observations for Tet2‐KD were unexpected and are in contrast to published Tet2‐KO models, similarly to the Tet‐TKD/Tet‐TKO discrepancy, with Tet2‐KO mice derived from Tet2^+/−^ or Tet2^−/−^ crosses (Ko *et al*, 2011; Li *et al*, 2011). For Tet2, we were not able to test KD efficiency via immunostaining as we did for Tet1 and Tet3 (see Fig EV2A and C); likely because Tet2 expression levels are low in mouse oocytes and early embryos (Wossidlo *et al*, 2011). Thus, to validate the phenotype for Tet2, we analyzed a second, non‐overlapping MO (Tet2‐MO2, see Appendix Table S1). This recapitulated the developmental phenotypes of the first MO (Fig EV1C). Moreover, we further verified the Tet2‐KD mediated arrest in rescue experiments to exclude potential off‐target effects of the Tet2‐MO. To this end, we co‐injected *in vitro*‐transcribed Tet2‐mRNA along with Tet2‐MO2, whose target sequence is not included in the injected mRNA sequence (Appendix Fig S1A and C). As controls, we co‐injected control‐MO together with Tet2‐mRNA and Tet2‐MO2 together with GFP‐mRNA (Fig 1E). The control group developed similar to non‐injected embryos (Fig EV1B) and embryos co‐injected with Tet2‐MO2 and GFP‐mRNA resulted in arrested preimplantation embryos similar to Tet2‐MO2‐injected embryos (Fig 1E). Importantly, co‐injection of Tet2‐MO2 with Tet2‐mRNA significantly rescued the Tet‐MO2 phenotype, with 27% of the embryos developing to the blastocyst stage (Fig 1E). Thus, our rescue experiments validated the specific phenotype we observed in Tet2‐KD embryos, which differs from the reported phenotype in Tet2‐KO mice which develop to term and are fertile (Ko *et al*, 2011; Li *et al*, 2011).

In summary, our analysis of Tet‐KD experiments revealed a striking early developmental arrest of Tet‐TKD embryos primarily at the 2‐cell stage upon acute depletion of Tet enzymes in the oocyte‐to‐embryo transition, with the most severe phenotype linked to Tet2. In contrast to Tet‐KO models, our KD studies demonstrate the essential role of Tet enzymes during preimplantation development.

### Tet1/2 generates 5hmC and 5caC in the zygote

**Figure 2:**
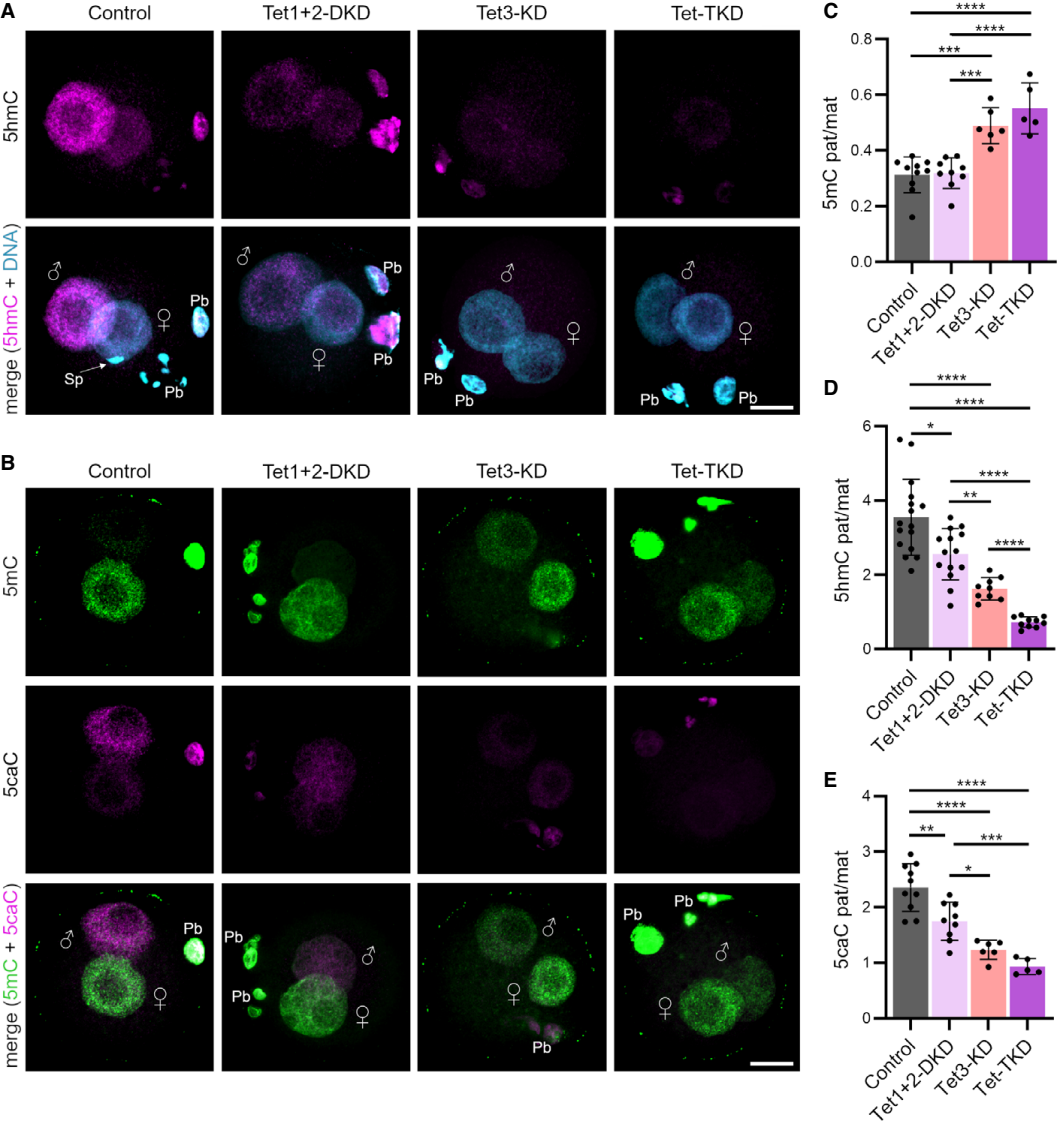
Tet enzymes have distinct roles in DNA methylation reprogramming in the mouse zygote A, BRepresentative images of (A) 5hmC‐ and (B) 5mC and 5caC‐IF of control (control‐MO injected), Tet1 + 2‐DKD and Tet‐TKD zygotes at 12 hpf. Paternal and maternal pronuclei are indicated, Pb = polar body; Sp = sperm; scale bar = 20 μm.C–EQuantification of paternal/maternal (C) 5mC‐, (D) 5hmC‐, and (E) 5caC‐signal ratios of 12 hpf derived zygotes normalized against DNA signal. A total of 5–16 zygotes from two to three experiments per condition were analyzed. Significance to control zygotes was calculated using ordinary one‐way ANOVA with Tukey's multiple comparisons test (C, E) or Welch ANOVA with Dunnett's T3 multiple comparisons test (D) and significant differences are indicated (*P < 0.05, **P < 0.01, ***P < 0.001, ****P < 0.0001, data are represented as mean ± SD, with dots representing single zygotes).

Tet3 was the only family member of Tet enzymes shown to play an important role in the conversion of 5mC to 5hmC in the zygote, with decreased 5hmC levels in Tet3‐depleted zygotes (Gu *et al*, 2011; Wossidlo *et al*, 2011; Shen *et al*, 2014), and Fig EV2B). Given the observed phenotypes of the single Tet enzyme KDs, we questioned whether Tet enzymes function interchangeably or whether they possess non‐redundant roles in the enzymatic conversion of 5mC to 5caC. Therefore, we analyzed Tet1 + 2‐DKD, Tet3‐KD, and Tet‐TKD‐derived zygotes in G2‐phase (12 h post‐fertilization, hpf) by 5mC‐, 5hmC‐, and 5caC‐ immunostainings (Fig 2).

As expected, control zygotes showed a loss of 5mC‐ and gain of 5hmC‐/5caC‐signals in the paternal pronucleus compared to the maternal pronucleus ([Fig 2A–E], and (Inoue *et al*, 2011; Wossidlo *et al*, 2011)). Loss of 5mC in the paternal pronucleus of Tet1 + 2‐DKD zygotes is similar to control zygotes, indicating functional enzymatic oxidation of 5mC by Tet3 in Tet1 + 2‐DKD zygotes and highlighting the important role of Tet3 in active DNA demethylation of 5mC. In contrast, Tet3‐KD and Tet‐TKD zygotes showed a reduced loss of 5mC‐signal ratios between paternal/maternal pronuclei (Fig 2B and C), suggesting a loss of 5mC by passive DNA demethylation only during S‐phase in these zygotes. This reduced loss of 5mC goes along with the complete abolishment of 5hmC‐ and 5caC‐signals in Tet‐TKD zygotes (Fig 2A and B). Notably, Tet1 + 2‐DKD zygotes showed decreased levels of 5hmC‐ and 5caC‐ratios compared to the control group, but still higher levels compared to Tet3‐KD or Tet‐TKD zygotes (Fig 2A, B, D and E). Interestingly, the Tet3‐KD showed a significant higher loss of 5hmC‐ratios compared to control and Tet1 + 2‐DKD zygotes, but still higher ratios compared to the Tet‐TKD (Fig 2A and D).

While these data confirm the important role of Tet3 in the early phase of zygotic DNA demethylation, they also indicate that the enzymatic oxidation of 5mC to subsequently 5caC in the mouse zygote is not only dependent on Tet3 activity but also in part on Tet1/Tet2. This points to non‐redundancy and a specific role for individual Tet enzymes in the stepwise oxidation of 5mC during this phase of DNA methylation reprogramming. Specific contributions of Tet enzymes in the oxidation steps of 5mC were also recently reported during the differentiation of mESCs (Mulholland *et al*, 2020). As 5hmC and 5caC are persistent DNA modifications in the mouse 2‐cell embryo (Inoue *et al*, 2011; Wossidlo *et al*, 2011) and Tet1 + 2 DKD embryos did not develop to the blastocyst stage, our results suggest important functions of specific DNA modifications in early preimplantation development. Factors that bind to 5hmC, 5fC, and 5caC are only beginning to be identified (Yildirim *et al*, 2011; Iurlaro *et al*, 2013; Song *et al*, 2013; Spruijt *et al*, 2013; Hashimoto *et al*, 2015; Nanan *et al*, 2019), and the function of these modifications regarding EGA and embryogenesis still remains unclear.

### Tet‐TKD 2‐cell embryos are characterized by a transcriptional signature of pre‐EGA embryos and cannot complete EGA

In mouse, the major wave of EGA occurs at the late 2‐cell stage, and embryos failing to perform EGA do not develop beyond 2‐cell stage (Aoki *et al*, 1997; Hamatani *et al*, 2004). Our findings that the vast majority of Tet‐TKD embryos arrest at the 2‐cell stage provided support for the concept that Tet enzyme function impacts EGA. Hence, we hypothesized that Tet enzymes and conversion of 5mC to 5caC are essential prerequisites for EGA. To test this hypothesis, we analyzed Tet‐TKD and Tet3‐KD‐derived 2‐cell embryos at G2‐stage by single‐embryo RNA‐Seq (Dataset EV1). In addition, we also performed single‐embryo RNA‐seq on α‐amanitin‐treated control‐MO derived 2‐cell embryos, which selectively inhibits RNA polymerase II/III, to obtain a list of EGA genes (Dataset EV2).

**Figure 3:**
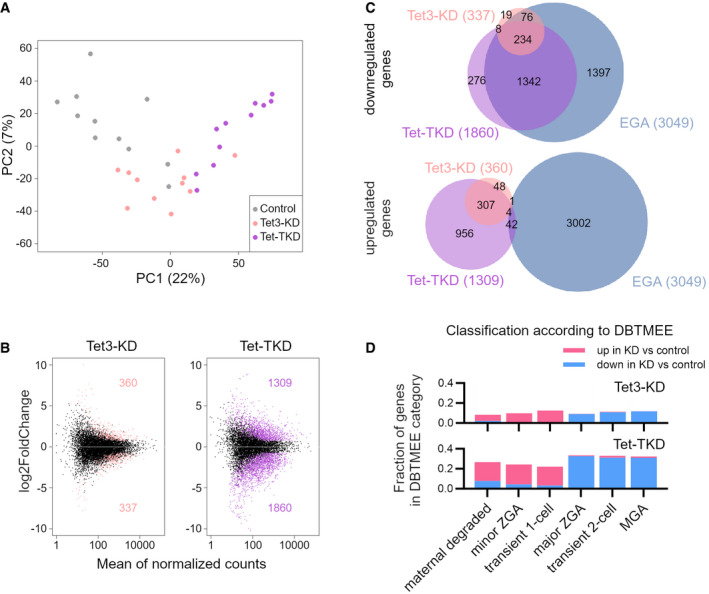
Tet enzymes are required for completion of embryonic genome activation Principal component analysis of RNA‐Seq data of genes from single Tet‐TKD (purple), Tet3‐KD (coral), and control (gray) 2‐cell embryos. Each dot represents a single 2‐cell embryo (control *n* = 11, Tet3‐KD *n* = 11, Tet‐TKD *n* = 11).MA plots of RNA‐seq data from Tet3‐KD/control (left) and Tet‐TKD/control (right) 2‐cell embryos. Significant differentially expressed genes are colored. The amount of up‐ and downregulated genes is indicated. Differentially expressed genes were determined using DEseq2 (*P*adj < 0.05, |log_2_FC| > 0.7).Venn diagrams visualizing the overlap of differentially expressed genes in Tet3‐TKD, Tet‐TKD, and embryonically activated genes (EGA) in 2‐cell embryos. EGA genes were calculated with DEseq2 comparing alpha‐amanitin‐treated and control 2‐cell embryos (*P*adj < 0.05, log_2_FC > 0.7).Comparison of transcriptional profiles of Tet3‐KD and Tet‐TKD 2‐cell embryos to a published database of early embryonic transcripts (DBTMEE, see (Park *et al*, 2015)). Shown are overlaps of DBTMEE categorized genes with the differentially expressed genes in Tet3‐KD or Tet‐TKD embryos compared with control 2‐cell embryos.

Principal component analysis (PCA) of RNA‐Seq results revealed segregation of Tet‐TKD from control embryos along PC1, with Tet3‐KD embryos clustering between the two samples (Fig 3A). 1,860 genes were downregulated in Tet‐TKD 2‐cell embryos, whereas only 337 genes were significantly decreased in Tet3‐KD embryos (Fig 3B). 1,309 and 360 genes were upregulated in Tet‐TKD and Tet3‐KD embryos, respectively. Notably, when comparing differentially expressed genes to EGA genes, the vast majority of genes downregulated in Tet‐TKD and Tet3‐KD overlapped with EGA genes, 1,576 of 1,860 (85%) and 310 of 337 (92%), respectively (Fig 3C), while upregulated genes are depleted for EGA genes, indicating a significant contribution of Tet enzymes to EGA. In total, Tet‐TKD embryos failed to activate 52% of EGA genes (1,576 of 3,049), whereas Tet3‐KD had a minor effect on EGA genes (12%, 381 of 3,049). These findings were further corroborated by comparing hierarchical clustering of differentially expressed genes, in which Tet‐TKD 2‐cell embryos clustered with transcriptionally inhibited α‐amanitin embryos and Tet3‐KD with control‐MO injected embryos (Appendix Fig S2A and B). Together, these results demonstrate that Tet‐TKD embryos are deficient in completely activating the embryonic genome.

**Figure EV3:**
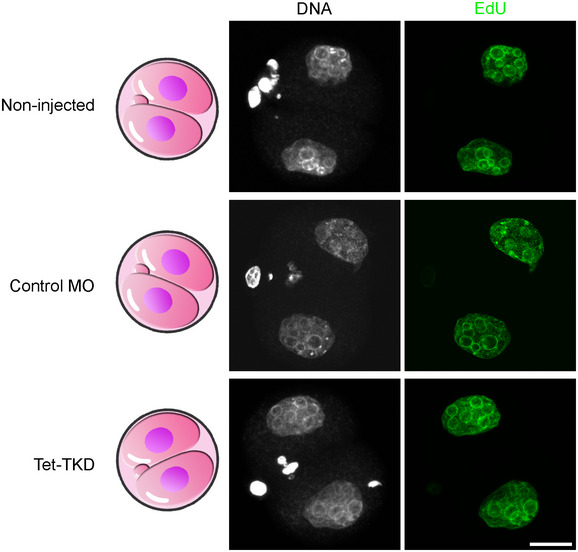
Tet‐TKD embryos undergo S‐phase in the 2‐cell stage Representative images of 2‐cell embryos derived from non‐injected, control‐morpholino, or Tet‐1–3 MO injected GVOs, which were incubated with EdU from 22.5 hpf until 27.5 hpf and analyzed for EdU incorporation. All three groups show similar incorporation of EdU indicating that Tet‐TKD 2‐cell embryos undergo replication in the 2‐cell stage (Tet‐TKD: *n* = 5, non‐injected: *n* = 3, control‐MO: *n* = 2; scale bar = 20 µm).

Next, we compared transcription profiles of Tet3‐KD and Tet‐TKD 2‐cell embryos with a public database of early mouse embryonic transcriptomes (DBTMEE (Park *et al*, 2015)). This comparison revealed a clear transcriptional signature for an arrest of Tet‐TKD embryos before the major wave of EGA (Fig 3D) and shows that genes, which are detected as upregulated in KD embryos, are in the majority transcripts which are maternally degraded or activated during minor EGA in control embryos. The transcriptome of Tet3‐KD embryos revealed only a minor arrest signature, which was consistent with the only partly impaired developmental progression of Tet3‐KD embryos (Fig 3D). Importantly, the transcriptional arrest of Tet‐TKD embryos before the major wave of EGA is independent of cell cycle progression, as Tet‐TKD embryos still undergo replication at the 2‐cell stage (Fig EV3).

We performed gene ontology (GO) analysis of genes that are less abundant in Tet3‐ and Tet‐TKD 2‐cell embryos and found highly significant GO processes enriched for terms like “RNA metabolic process, RNA processing, ncRNA processing, translation”, with in general lower *P*‐values for Tet3‐KD than Tet‐TKD embryos (Table EV1). Additionally, genes downregulated in Tet‐TKD embryos were enriched for GO processes like “metabolic process, mRNA processing, RNA splicing, cell cycle”, mirroring the developmental phenotype of Tet‐TKD embryos and suggesting failures in establishing biological processes that are vital for early development.

When performing ChIP Enrichment Analysis for downregulated genes, we found many prominent transcription factors and epigenetic modifiers essential for early development (Appendix Table S2). Most enriched factors appeared in both Tet3‐KD and Tet‐TKD downregulated gene sets. Targets of Tet1, the only present Tet enzyme in the reference dataset, were detected as significantly enriched in Tet‐TKD embryos only. For this analysis, we analyzed published datasets from closely related mouse embryonic stem cells (mESCs) (Lachmann *et al*, 2010; Kuleshov *et al*, 2016), as ChIP‐Seq experiments in preimplantation embryos are technically not feasible yet. This comparison to mESCs suggests that downregulated genes in Tet‐TKD are, at least partially, direct targets of Tet enzyme activities; however, as Tet enzymes target DNA not directly, these correlation analyses need to be interpreted with caution.

### Tet enzyme‐depleted embryos dysregulate transposable elements and do not activate MERVL‐driven genes

**Figure 4:**
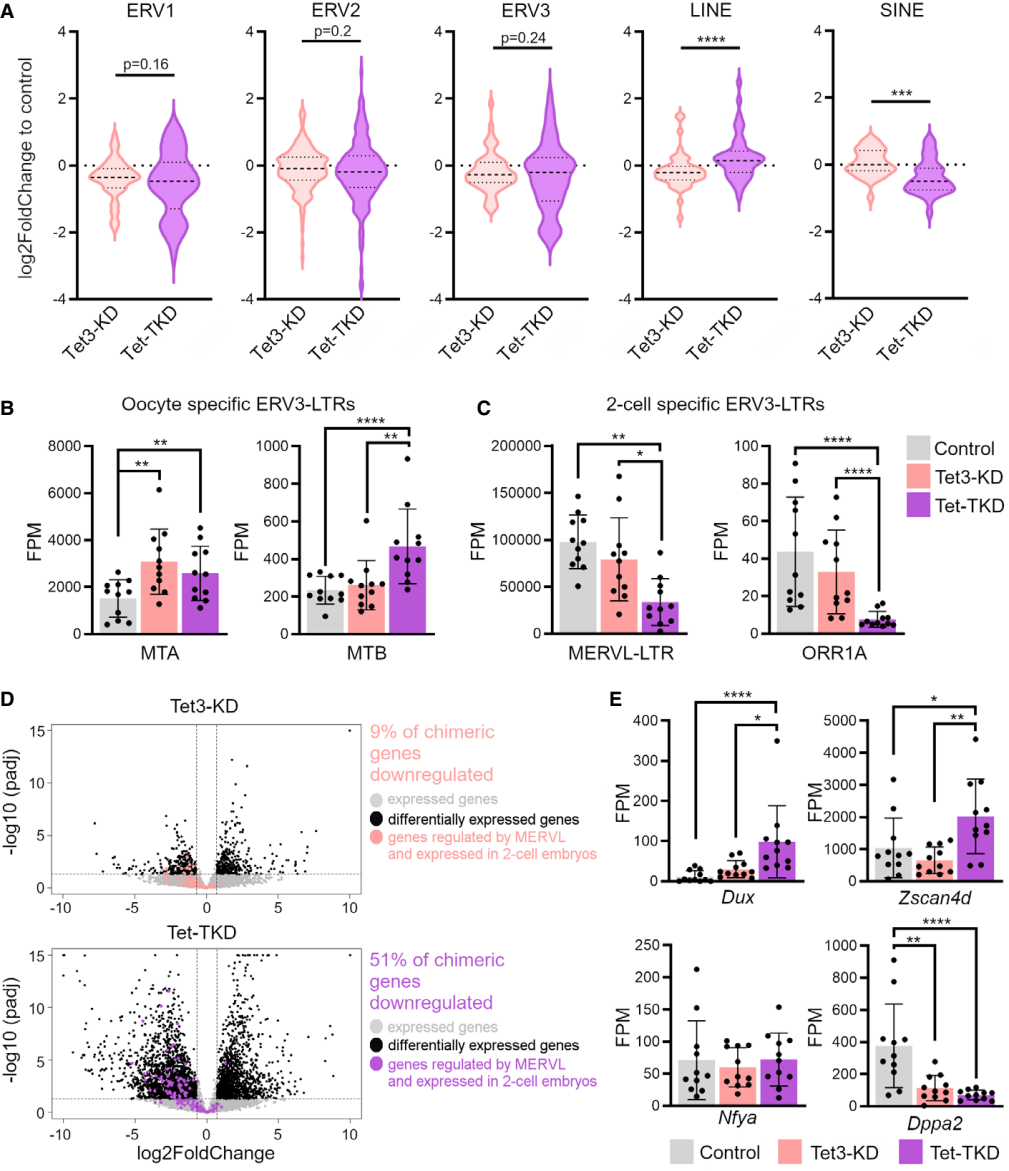
Tet enzyme deficiency dysregulates specific classes of transposable elements (TEs) and 2C genes AExpression changes of different classes of TEs in Tet3‐KD and Tet‐TKD. Shown are log_2_‐fold changes of KD embryos compared to control 2‐cell embryos of different repetitive elements subdivided into classes of TEs. Indicated *P*‐values were calculated using a paired two‐tailed t‐test (***P < 0.001, ****P < 0.0001, data are represented as medium smoothed violin plots with indicated median and quartiles as dotted lines).B, CExpression levels of (B) maternally expressed ERV3‐LTRs and (C) 2‐cell embryo specific ERV3‐LTRs in control, Tet3‐KD and Tet‐TKD 2‐cell embryos (*P*‐values were calculated using DEseq2; *P < 0.05, **P < 0.01, ***P < 0.001, ****P < 0.0001, data are represented as mean ± SD, dots represent single embryos).DVolcano plots showing expression changes of genes in Tet3‐KD and Tet‐TKD 2‐cell embryos. Chimeric genes, as defined by the presence of a nearby MERVL element and upregulation in 2‐cell embryos, are indicated (see (Macfarlan *et al*, 2012)). Dashed lines represent the cutoff for differential expression: |log_2_FC| > 0.7 and *P*adj < 0.05.EExpression levels of important candidate factors in control, Tet3‐KD and Tet‐TKD 2‐cell embryos, implicated in the generation of 2C‐like cells and as important regulators of EGA (*P*‐values were calculated using DEseq2; *P < 0.05, **P < 0.01, ****P < 0.0001, data are represented as mean ± SD, dots represent single embryos).

Since early mouse development is characterized by a transcriptional burst of (retro)transposable elements at the 2‐cell stage, we analyzed the impact of Tet enzyme depletion on the expression of transposable elements (TEs). PCA of TE expression levels showed a tendency for segregation of Tet‐TKD from control embryos along PC1, with Tet3‐KD embryos scattered in between both groups (Appendix Fig S3A). Overall, Tet‐TKD embryos were characterized by misregulation of many TEs, which was not as strongly pronounced in Tet3‐KD embryos (Fig 4A, and Appendix Figs S3B and S4, Dataset EV3). The class of long interspersed nuclear elements (LINEs) was slightly upregulated in Tet‐TKD embryos (Fig 4A), indicating that Tet enzymes were not required for the activation of LINE elements, as also shown before upon Tet3‐KD (Inoue *et al*, 2012). Notably, short interspersed nuclear elements (SINEs) were tendentially downregulated in Tet‐TKD embryos (Fig 4A and Appendix Fig S4). SINEs compromise about 8% of the mouse genome and are typically methylated to prevent transposition (Meissner *et al*, 2008). SINEs can cause hypermethylation of nearby genes (Estecio *et al*, 2012), which could play an important role for EGA and will need further investigation. Interestingly, long terminal repeat (LTR) elements showed up‐ and downregulation of specific class members (Fig 4A, and Appendix Fig S4). The class III of endogenous retroviruses (ERV3‐ including MERVL and MaLR elements) has been reported for its element‐specific differential expression during embryonic development (Franke *et al*, 2017) and ERV3 elements are regulated via different LTR sequences. Notably, ERV3‐LTRs that are expressed during late oogenesis showed higher expression levels in Tet‐TKD embryos compared to control embryos (MTA, MTB; Fig 4B) and ERV3‐LTRs, which are highly expressed specifically in two‐cell embryos, are significantly downregulated in Tet‐TKD embryos (MERVL‐LTR, ORR1a2; Fig 4C). These observations further corroborated a characteristic transcription profile of embryos arrested pre‐EGA and suggested that this developmentally important class of TEs are also regulated by Tet enzymes in the early embryo.

**Figure EV4:**
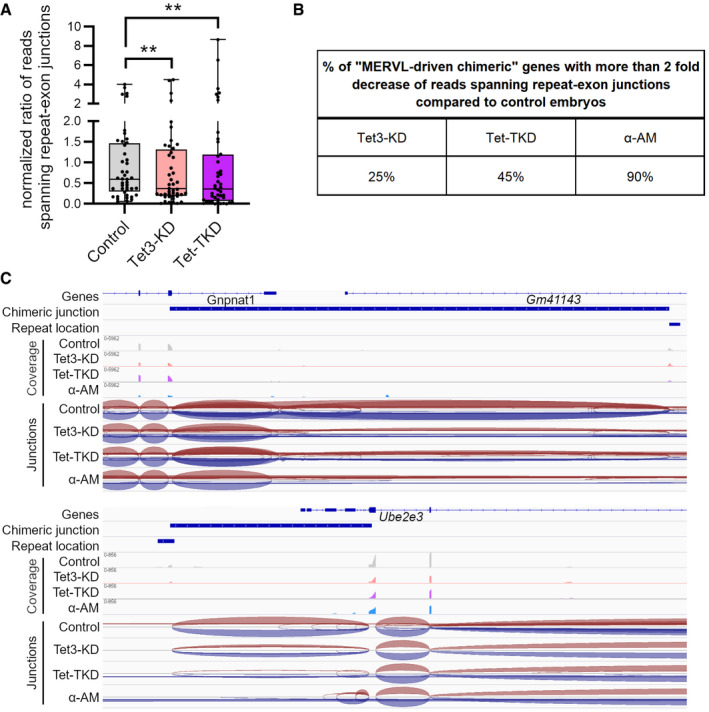
Expression analysis of MERVL‐driven chimeric transcripts Normalized ratio of reads spanning the repeat‐exon junctions of “MERVL‐chimeric” genes according to MacFarlan *et al* (2012). Statistical significance was tested using Friedman test with Dunn's multiple comparisons test (**P < 0.01). Data from all embryos of one condition (11 embryos each) were merged for this analysis. Data are represented as box plots with indicated mean and quartiles with whiskers representing the min and max values; dots represent different repeat‐exon junctions.Percent of analyzed “MERVL‐driven chimeric” genes, which show a 2‐fold decrease of the usage of the chimeric junction compared to control embryos. RNA‐seq data from all embryos of one condition were merged for this analysis. Note: In α‐Amatinin (α‐AM) 2‐cell embryos for 85% of genes no junctions could be observed (only genes which had more than 1,000 reads mapping to the whole transcript were considered).Screenshots of IGV genome browser showing RNA‐seq data of control, Tet3‐KD, Tet‐TKD, and α‐AM treated 2‐cell embryos at chimeric repeat‐exon junction sites of the “2C‐genes” Gnpnat1 and Ube2e3.

MERVL elements have been reported to facilitate the expression of a set of genes during EGA—often as new chimeric transcripts using their LTR sequences as alternative promoters (Macfarlan *et al*, 2012). Remarkably, 51% of MERVL‐driven genes, which were shown to be activated in 2‐cell embryos, were significantly downregulated in Tet‐TKD embryos (72 of 141 detected genes, Fig 4D; see (Macfarlan *et al*, 2012), Table EV2). In contrast, only 9% of MERVL‐driven genes were significantly downregulated in Tet3‐TKD embryos (12 of 141, Fig 4D). In addition, hierarchical clustering for chimeric genes revealed the misregulation of these genes in Tet‐TKD embryos and to a lower extent also in Tet3‐KD embryos (Appendix Fig S5). We also observed a significant decrease of chimeric junction usage in MERVL‐driven chimeric genes in Tet3‐KD and Tet‐TKD compared with control 2‐cell embryos (Fig EV4A–C, Table EV3).

Recently, several factors have been reported as master regulators to induce EGA and the expression of MERVLs. While quite a few factors are known to activate the expression of MERVL elements and “2C genes” in mESCs, which induce a so‐called “2C‐like” state (Macfarlan *et al*, 2012; Eckersley‐Maslin *et al*, 2019), two prominent transcription factors, Dux and Nfya, stand out as potential master regulators or pioneering factors in 2‐cell embryos. Three studies suggested that *Dux*, an early‐induced gene in mouse preimplantation development, acts as a pioneering factor activating promoter regions of MERVL elements and 2C genes (De Iaco *et al*, 2017; Hendrickson *et al*, 2017; Whiddon *et al*, 2017). Interestingly, *Dux* expression was upregulated in Tet‐TKD embryos (Fig 4E). While some targets of Dux, like Zscan4, a prominent factor expressed in 2C‐stage embryos and a regulator of the 2C‐state in mESCs (Rodriguez‐Terrones *et al*, 2018; Srinivasan *et al*, 2020), were still activated in Tet‐TKD embryos, the MERVL‐driven chimeric genes were not activated to full levels (Fig 4D and E). Next to this, the maternal factor Nfya, which is implicated in chromatin opening during the early phase of EGA (Lu *et al*, 2016), did not show expression changes in Tet‐TKD embryos (Fig 4E). In contrast, Dppa2 and Dppa4, whose overexpression induces the expression of MERVL elements and the 2C‐state in mESCs via Dux (Eckersley‐Maslin *et al*, 2019), were highly downregulated in Tet‐TKD and Tet3‐KD embryos (Fig 4E). Taken together, our results suggest that epigenetic modifiers like Tet enzymes, in combination with the activity of several key transcription factors, are critical for generating a transcriptionally permissive state before activating genes during EGA.

Our findings on the impact of Tet enzymes on EGA are in line with reported *Stella* mutant phenotypes in the oocyte‐to‐embryo transition. Stella (alias PGC7) is protecting the maternal pronucleus from Tet enzyme activity (Nakamura *et al*, 2012) and DNA *de novo* methylation mediated by Uhrf1/Dnmt1 (Li *et al*, 2018). Stella‐deficient embryos show higher DNA methylation levels and are characterized by abnormal preimplantation development similar to Tet‐TKD embryos; they are impaired in the oocyte‐to‐embryo transition (Li *et al*, 2018), rarely reach the blastocyst stage (Payer *et al*, 2003), and show many misregulated TEs similar to Tet‐TKD 2‐cell embryos (Huang *et al*, 2017). Together, these data suggest that a fine‐orchestrated balance of DNA methyltransferases and Tet enzymes is needed in germ cells and early preimplantation development for the successful completion of EGA.

### Tet enzymes have a minor contribution to genome‐wide DNA demethylation suggesting the involvement of non‐catalytic functions of Tet enzymes

**Figure EV5:**
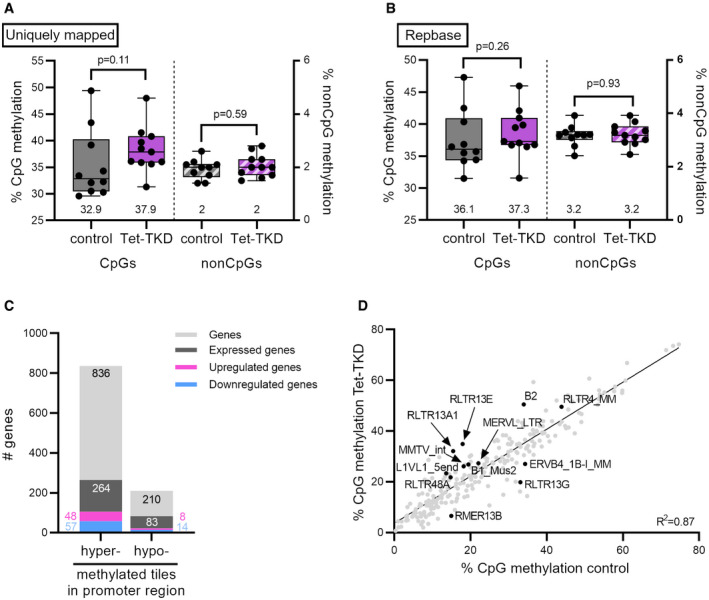
BS‐Seq analysis of control and Tet‐TKD 2‐cell embryos A, BOverall methylation levels of cytosines in CpG context and non‐CpG context in (A) uniquely mapped reads or (B) reads mapping to reference sequences obtained from Repbase (Bao *et al*, 2015). Numbers indicated below the box plot represent the median. Data is represented as box plots, with indicated median and quartiles with whiskers representing the min and max values; dots represent single 2‐cell embryos (control *n* = 10, Tet‐TKD *n* = 11). Statistical significance was tested using Mann–Whitney test.CNumber of genes associated with hypo‐ or hypermethylated 500‐bp tiles in their promoter region (TSS −5 kb/+1 kb).DCorrelation of CpG methylation of repetitive elements in control and Tet‐TKD 2‐cell embryos. Indicated are significantly differentially methylated genes with a methylation difference of > 5% and an adjusted *P*‐value < 0.05. % CpG methylation corresponds to the methylation levels in the merged datasets of each condition over the annotated repeat element.

Next, we questioned whether the observed expression changes can be correlated to DNA methylation changes in Tet‐TKD embryos. Therefore, we performed whole‐genome bisulfite sequencing (BS‐seq) on single control‐ and Tet‐TKD 2‐cell embryos. Overall, we found only minor changes in total CpG methylation levels (32.9% in control vs 37.9% median CpG methylation in Tet‐TKD, Fig EV5A), similar to reported total methylation changes upon Tet3‐KO in zygotes (Peat *et al*, 2014).

**Figure 5:**
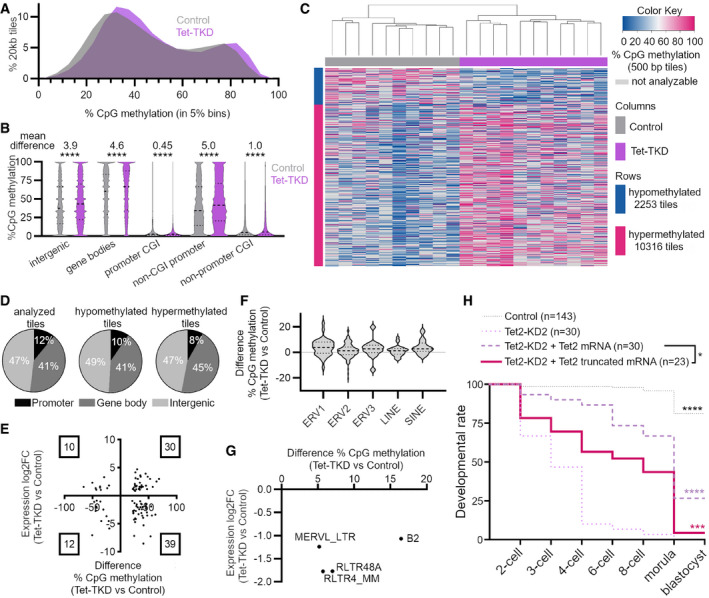
Minor impact of Tet enzymes on genome‐wide DNA demethylation and partial rescue of developmental arrest by non‐catalytical functions % CpG methylation of 20‐kb tiles in 5% bins in control or Tet‐TKD 2‐cell embryos. All samples from one condition were merged for this visualization.% CpG methylation of 500‐bp tiles for different genomic features. All samples from one condition were merged for this visualization. Intergenic = genome excluding gene bodies and TSS −5 kb/+1 kb; gene bodies = genes excluding TSS −5 kb/+1 kb; promotor‐CGI = CGIs overlapping TSS −5 kb/+1 kb; non‐CGI promoter = promoter (TSS −5 kb/+1 kb) not close to CGI; non‐promoter CGI = CGIs not overlapping TSS −5 kb/+1 kb. (*P*‐values were calculated using a Wilcoxon matched‐pairs signed rank test and presented in a medium smoothed violin plot with indicated median and quartiles as dotted lines, ****P < 0.0001; mean difference is indicated above the plot).Heatmap of differentially methylated uniquely mapped 500‐bp tiles. Shown are random 20% of all significantly hypo‐ (450 of 2253) and hypermethylated (2,063 of 10,316) tiles (logistic regression function of Seqmonk, *P*adj < 0.01, difference 20%, for details see Materials and Methods section).Distribution of differentially methylated tiles over promoter, gene bodies, or intergenic sequences.Correlation of significant DNA methylation changes in promoter regions and differentially expressed genes. Numbers in boxes represent number of genes.Significant methylation changes of specific TEs subdivided in different classes of TEs (calculated using logistic regression function of Seqmonk, *P*adj < 0.05; data are represented as medium smoothed violin plot with indicated median and quartiles as dotted line).Correlation of significant DNA methylation changes in TEs (calculated with logistic regression function of Seqmonk, *P*adj < 0.05, methylation difference > 5%), which are also significant differentially expressed (DEseq2, *P*adj < 0.05, |log_2_FC| > 0.7).Developmental rate of embryos derived from control, Tet2‐MO2, Tet2‐MO2 + Tet2 full‐length mRNA (as shown in Fig 1E) and Tet2‐MO2 + Tet2 truncated mRNA (no catalytical domain) injected oocytes. Indicated *P*‐values were calculated using the log rank (Mantel‐Cox) test against Tet2‐MO2 KD or as indicated (*P < 0.05, ***P < 0.001, ****P < 0.0001, numbers of analyzed embryos for each condition is indicated in parenthesis).

Non‐CpG methylation is present in oocytes at around 3.5% of non‐CpG positions, which is then decreasing over early cleavage stages (Shirane *et al*, 2013). Accordingly, we detected non‐CpG methylation at a median level of 2% with no significant difference between both groups (Fig EV5A). Next, we analyzed methylation levels in 20,000‐bp tiles and found a general shift toward higher methylated tiles in Tet‐TKD embryos (Fig 5A). Analyzing 500‐bp tiles, we found that those changes can be observed genome‐wide without a preference for a particular genomic feature (Fig 5B). When analyzing significant differentially methylated 500‐bp tiles, we found 10,316 hypermethylated and 2,253 hypomethylated tiles (Fig 5C). Hypermethylated tiles seem to be slightly enriched in gene bodies (Fig 5D). 836 and 210 gene promoters (TSS −5 kb/+1 kb) are associated with hyper‐ and hypomethylated tiles, respectively (see Dataset EV4). Gene ontology analysis of these genes did not reveal vital biological processes. We also did not observe a direct correlation of BS‐Seq data to gene expression data of Tet‐TKD embryos (Figs 5E and EV5C, and Appendix Fig S6).

Since we observed changes in the expression of TEs in Tet‐TKD embryos, we also analyzed the methylation status of TEs in our dataset. Here, while the overall methylation levels of TEs are similar, we detected significant changes in CpG methylation of specific TEs in Tet‐TKD embryos (Fig EV5B and D, Dataset EV5). These differentially methylated TEs were found in all classes of retrotransposons and the majority showed an increase in methylation levels in Tet‐TKD embryos (Fig 5F). Interestingly, when correlating expression changes of TEs and their corresponding DNA methylation changes in Tet‐TKD embryos vs control embryos, we found the ERV3 class member MERVL‐LTR, the SINE class member B2, and two ERV1 class members with higher DNA methylation levels and decreased expression levels (Fig 5G). Although this correlation of DNA methylation and gene expression changes of MERVL‐LTRs let us speculate that specific chimeric transcripts are directly regulated via the change of DNA methylation levels of nearby MERVL‐LTRs by Tet enzymes, we did not observe a direct correlation of expression changes of chimeric genes and the methylation level of associated repeats (Appendix Fig S7A and B). However, as BS‐Seq cannot discriminate between 5mC and 5hmC, and as 5hmC is present around 5–10% in early preimplantation embryos (see (Amouroux *et al*, 2016)) and a persisting DNA modification in early preimplantation development (Inoue & Zhang, 2011), further studies are needed to decipher the genomic regions of 5hmC and its impact on early preimplantation development.

Recently, Tet enzymes have been shown to also possess non‐catalytical functions in recruiting chromatin modifiers and other factors to the genome, which can lead to gene expression changes (Xu *et al*, 2012; Kaas *et al*, 2013; Neri *et al*, 2013; Zhang *et al*, 2015; Montagner *et al*, 2016; Montalban‐Loro *et al*, 2019). These alternative functions of Tet enzymes could also explain the observed phenotypes in our Tet‐KD studies compared to the normal preimplantation development in catalytic mutants reported in germline Tet‐KO studies, especially since we observed only minor changes of DNA methylation in Tet‐TKD embryos and as we did not find an obvious enrichment of methylation changes in the promoter region of differentially expressed genes, which are shown to be important at this developmental stage. Thus, we next analyzed whether the catalytical activity of Tet2, which we identified as most crucial during early stages during development, is needed at this early embryonic stage or whether there might be non‐catalytical functions involved. We designed a rescue experiment for Tet2 using a truncated Tet2 mRNA mimicking the catalytical KO of Tet2 in previous germline Tet‐TKO study (Dai *et al*, 2016) (Appendix Fig S1B and D). While truncated expression products of genes can mediate non‐sense mediated decay, as discussed as a complication in KO approaches (Wilkinson, 2019), or lead to disruptions of protein–protein interactions, a shorter isoform of Tet2 (missing the catalytical domain) is expressed in human oocytes and early human cleavage‐stage embryos, suggesting an important non‐catalytic function of the C‐terminal part of Tet2 (Hendrickson *et al*, 2017). Strikingly, co‐injection of the truncated Tet2 mRNA in Tet2‐MO2‐injected oocytes partially rescued preimplantation development (Fig 5H), indicating that non‐catalytical functions of Tet2 might play at least in part a critical role during the oocyte‐to‐embryo transition and which will need further investigations on the molecular mechanism of Tet2 activity, in particular on the activation of TEs.

To further add to the complexity of Tet enzyme biology in early development, recent studies have highlighted the contribution of Tet enzymes in remodeling RNA methylation with implications for post‐transcriptional gene regulation and RNA stability (Guallar *et al*, 2018; Shen *et al*, 2018; Lan *et al*, 2020; He *et al*, 2021), which could play important functions in EGA and the degradation of maternally stored RNAs and should be addressed in future studies.

In conclusion, our results demonstrate that Tet enzymes are a critical component of the regulatory clock required to complete EGA in mammalian embryos, which suggests an important function of Tet enzymes in the establishment of a permissive chromatin landscape, allowing the generation of totipotent and pluripotent embryonic cells. Our findings in combination with other studies (Payer *et al*, 2003; Lu *et al*, 2016; De Iaco *et al*, 2017, 2020; Hendrickson *et al*, 2017; Whiddon *et al*, 2017; Guo *et al*, 2019) indicate that EGA occurs in a highly orchestrated and multifactorial process, including several layers of chromatin reprogramming together with specific regulation by key transcription factors, rather than an all‐or‐none program.

## Materials and Methods

### Animal experiments

All mouse experiments were carried out in accordance with the Stanford University Administrative Panel on Laboratory Animal Care and the Medical University Vienna in agreement with the authorizing committees.

### Isolation, microinjection of MOs, *in vitro* maturation and *in vitro* fertilization of germinal vesicle oocytes

The KD strategy of Tet enzymes in mouse oocytes is outlined in Fig 1A. As KD approach, morpholinos were chosen, since they provided a more efficient knockdown compared to siRNAs of Tet enzymes (for Tet3 compare this study to (Wossidlo *et al*, 2011)). Briefly, 48‐h post‐injection of 5 IU pregnant mare serum fully grown oocytes at the germinal vesicle stage were obtained from ovaries of 6–8 weeks old F1(C57BL/6×DBA/2) female mice and transferred to M2 medium without BSA (Nagy, 2003), supplemented with 0.2 mM 3‐isobutyl‐1‐methylxanthine (IBMX; Sigma‐Aldrich). After 10 min of Hoechst 33342 (0.05 μg/ml) incubation, SN‐type GVOs were microinjected in different combinations with MOs designed against Tet1–3 enzymes (Gene Tools, 100 μM, see Appendix Table S1), co‐injected with dextran‐tetramethyl‐rhodamine (Invitrogen, 3,000 MW, 100 μg/ml) to monitor injection volumes for reproducible microinjections. As negative control, a widely used standard control‐MO targeting the human β‐globin pre‐mRNA was used in concentrations matching the molarity of single, double, or triple combined MOs used in respective experimental groups. For rescue experiments, Tet2‐MO2 was co‐injected with either *in vitro*‐transcribed full‐length Tet2‐mRNA or truncated Tet2‐mRNA, missing the catalytic domain, in 100 ng/µl concentrations (see detailed below). MO‐injected GVOs were washed in IMBX free α‐MEM medium supplemented with 5% FBS and 10 ng/ml EGF and incubated in a humidified atmosphere with 6% CO_2_, 5% O_2_, and 89% N_2_ at 37°C for 16–18 h to complete meiotic maturation. Spermatozoa collection and IVF procedures were carried out as described (Nagy, 2003). Briefly, sperm was isolated from the *cauda epididymis* of adult F1(C57BL/6×DBA) male mice and capacitated by pre‐incubation for 1.5 h in pre‐gassed HTF medium. *In vitro* matured oocytes were collected 16–18 h post‐injection of MOs. Mature MII oocytes were placed into a 100 μl drop of KSOMaa medium (Cytospring, Sigma‐Aldrich) with capacitated sperm and incubated at 37°C in a humidified atmosphere of 6% CO_2_, 5% O_2_, and 89% N_2_. Zygotes were analyzed by time‐lapse imaging or collected at distinct time points for further analysis. For the analysis of embryonically activated genes control‐MO injected, *in vitro* matured and fertilized zygotes at 4 hpf were cultured with 0.1 mg/ml α‐amanitin (Sigma‐Aldrich) and derived 2‐cell embryos were collected 29 hpf for RNA‐Seq analysis.

### Tet2 rescue experiments by co‐injection of full‐length Tet2 or truncated Tet2 mRNA

To generate Tet2 mRNA, full‐length Tet2 was amplified with a fusion‐primer including T7 promoter from FH‐Tet2‐pEF (a gift from Anjana Rao (Addgene plasmid #41710; http://n2t.net/addgene:41710; RRID:Addgene_41710), Appendix Fig S1A and C). For rescue experiments with mRNA coding for truncated Tet2, the plasmid was linearized with SpeI and amplified to obtain the sequence shown in Appendix Fig S1B and D, excluding the catalytic domain of Tet2. mRNA was then generated using mMESSAGE mMACHINE T7 Ultra Kit (Ambion) and purified using MEGAclear™ Transcription Clean‐Up Kit (Ambion) (Appendix Fig S1A and B). mRNA (100 ng/µl) was co‐injected with Tet2‐MO2 in GVOs as described above. As controls, control‐MO was co‐injected with 100 ng/µl full‐length Tet2 mRNA and Tet2‐MO2 co‐injected with 100 ng/µl GFP‐mRNA. Notably, the generated Tet2 mRNAs are not targeted by the Tet2‐MO2. After IVM and IVF, developmental potential until blastocyst stage was analyzed.

### Time‐lapse imaging of KD embryos

Mouse preimplantation development was monitored using a custom‐built miniature microscope system that was modified for non‐invasive darkfield illumination as previously described (Wong *et al*, 2010). Embryos were cultured in custom dishes with individual microwells to track single embryos during time‐lapse imaging, where all microwells shared one common media drop under mineral oil to maintain group culture. Images were taken every 5 min for up to 3.5 dpf (until the majority of control embryos reached the blastocyst stage). After each experiment, images were compiled into a time‐lapse movie by ImageJ.

### Embryo staining and immunofluorescence microscopy

For immunofluorescence analysis, embryos were briefly washed in M2 medium (Sigma‐Aldrich) and *zona pellucida* was removed by treatment with acidic tyrode's solution (Sigma‐Aldrich). Embryos were fixed for 20 min in 3.7% paraformaldehyde in PBS at 4°C and permeabilized with 0.2% Triton X‐100 in PBS for 10 min at RT. Fixed embryos were blocked overnight at 4°C in 1% BSA, 0.1% Triton X‐100 in PBS. Next, embryos were stained with anti‐Tet3 (rabbit polyclonal (1:500)), gift from Guo‐Liang Xu (Shanghai Institute of Biochemistry and Cell Biology, Chinese Academy of Sciences, Shanghai, China, see (Gu *et al*, 2011)) and anti‐H3K4me3 (mouse monoclonal [1:500], cat. no. 05‐1339, Millipore, CA USA), or anti‐Tet1 (rabbit polyclonal [1:100], cat. no. GTX125888, GeneTex, Irvine, CA USA). For 5mC‐, 5hmC‐, and 5caC immunostainings, fixed embryos were incubated in 4N HCl solution at RT for 15 min. Following neutralization (10 min, 100 mM Tris–HCl, pH 8.0) and second fixation, embryos were stained with anti‐5mC (mouse monoclonal [1:100], cat. no. BI‐MECY‐0100, Eurogentec, Fremont, CA USA), anti‐5hmC (rabbit polyclonal [1:100], cat. no. 39769, Active motif, Carlsbad, CA USA), or anti‐5caC (rabbit polyclonal [1:500], gift from Yi Zhang (Howard Hughes Medical Institute, Boston Children's Hospital, Boston, MA USA), see (Inoue *et al*, 2011)). Followed by several washes in blocking solution, embryos were incubated at RT with anti‐mouse or anti‐rabbit secondary antibodies for 2 h coupled with Alexa Fluor 488 (10 μg/ml) or 647 (10 μg/ml; ThermoFisher Scientific). After incubation in propidium iodide (10 min, 2 μg/ml), embryos were washed and mounted on slides with a small drop of Vectashield (VectorLab, Burlingame, CA USA) mounting medium. For the EdU incorporation assay, 2‐cell embryos were incubated in 50 µM EdU from 22.5 hpf until fixation with 3.7% PFA in PBS at 27.5 hpf. Fixed embryos were permeabilized with 0.2% Triton X‐100 in PBS for 10 min at RT. EdU click‐IT reaction was performed according to manufacturer's guidelines (ThermoFisher Scientific). Embryos were analyzed on a Zeiss LSM510 Meta inverted laser scanning confocal microscope or a Leica DMI 600 B fluorescent microscope as described (Wossidlo *et al*, 2010). ImageJ software was used to quantify antibody signals of z‐stack computed IF images (˜ 15–25 stacks with 0.5 μm per sample; for quantification of antibody signals, sum projections of single pronuclei were compiled to calculate the “corrected total cell fluorescence” (CTCF) according to a GitHub protocol by Martin Fitzpatrick (Queensland Brain Institute, University of Queensland, Australia; see https://github.com/mfitzp/theolb/blob/master/imaging/measuring‐cell‐fluorescence‐using‐imagej.rst).

### RNA‐Seq analysis

At 29 hpf, *zona pellucida* was removed from 2‐cell embryos, and individual embryos were collected for RNA‐Seq library construction. cDNAs were prepared with SMARTer Ultra Low RNA Kit (Clontech, Mountain View, CA USA) following manufacturer's protocol as previously described (Ramskold *et al*, 2012; Shalek *et al*, 2013). The cDNAs were amplified using Advantage 2 PCR Kit (Clontech, Mountain View, CA USA) and fragmented using Covaris S2 machine and Covaris microTUBEs (Covaris, Woburn, MA USA) as outlined in Clontech SMARTer Ultra Low RNA Kit protocol. The fragmented cDNAs were analyzed by Bioanalyzer High Sensitivity DNA kit (Agilent, Santa Clara, CA USA). 1 or 10 ng of amplified cDNAs was end‐repaired, dA‐tailed, ligated to adaptors, and amplified using NEBNext DNA Library Prep Master Mix Set for Illumina (New England BioLabs, Ipswich, MA USA). The libraries were sequenced 2 × 100 bases on Illumina HiSeq 2000, yielding approximately 6–26 M reads per sample. Reads were mapped to mouse reference genome mm10 with STAR2.5.1b using default settings. Counts of reads mapped to Refseq database, depleted for sex chromosome genes and with added locations of specific repeat sequences expressed in “MERVL‐driven chimeric transcripts” with corresponding exons (see (Macfarlan *et al*, 2012)), were obtained with HTseq‐count (v.0.13.5) (Anders *et al*, 2015). Genes that have at least 10 reads in 50% of the embryos of one condition were used for further analysis. Differentially expressed genes were obtained using DEseq2 (Love *et al*, 2014) (*P*adj < 0.05 and absolute log_2_FC > 0.7, Dataset EV1). For the analysis of *Dux* expression, reads were mapped with Hisat (v2.1.0) against AM398147.1 and counts were normalized using DEseq2 with single genes. EGA genes were defined as genes, which are significantly downregulated in α‐amanitin‐treated 2‐cell embryos vs control embryos (Dataset EV2). Counts of reads spanning junctions of repeat‐exon in MERVL‐chimeric transcripts (see (Macfarlan *et al*, 2012)) were determined using RegTools (preprint: Cotto *et al*, 2021) (Table EV3) and normalized by expression level of associated genes. To obtain expression levels of transposable elements, reads were mapped using Bowtie2 using default settings with –N 1 to annotations from Repbase (Bao *et al*, 2015), counts were obtained using HTseq (Anders *et al*, 2015) and normalized together with counts of single genes using DEseq2 (Love *et al*, 2014)(Dataset EV3). The Gene Expression Omnibus accession number for the RNA‐Seq data reported in this paper is GSE57063.

### Whole‐genome bisulfite sequencing

At 29 hpf, *zona pellucida* was removed from 2‐cell embryos, and individual control or Tet‐TKD 2‐cell embryos were collected for bisulfite library construction. As negative controls, media from the last washing step was used. For library preparation, a Post‐Bisulfite Adapter Tagging approach (PBAT) (Lee & Smallwood, 2018) was used with few changes: BD buffer (Qiagen) was used for desulphonation reaction, biotin‐capture was omitted and following oligos were used ‐ oligo 1: TCGTCGGCAGCGTCAGATGTGTATAAGAGACAGTNNNNNNNNN and oligo 2: GTCTCGTGGGCTCGGAGATGTGTATAAGAGACAGTNNNNNNNNN—to match the primers from the Nextera XT Index Kit, which were used for the library amplification in the last step using 12 cycles. Samples were sequenced first on a MiSeq for testing library quality. No cell controls were giving a mouse‐specific mapping rate of 0.001–4.2% and the samples on average 58.5%, confirming specificity of the sample libraries. Subsequently, 10 control and 11 Tet‐TKD libraries were pooled and sequenced on one high output NextSeq500/550 2× 100 bp run. Reads were trimmed using trim galore v0.4.2 and mapped to mm10 excluding all reads mapping to hg19 using Bismark v0.16.3 in single‐end mode (Krueger & Andrews, 2011). All mapped reads from each embryo were deduplicated and methylation values extracted using Bismark v0.16.3 (for statistics see Table EV4). Significant differentially methylated regions were determined of 500‐bp tiles using Seqmonk v.1.47.1 logistic regression function with minimum observations of three, minimum valid replicates of three with a *P*‐value cutoff of 0.01, and a methylation difference higher than 20%. To consider all replicas, a filter using a mean difference of more than 20% of 500‐bp tiles was applied (minimum three observations, minimum one count per position in Seqmonk settings) of all replicas and more than 20% methylation difference when treating all replicas from one condition as a single sample. GREAT 4.0.4. (McLean *et al*, 2010) was used to find genes with promoter regions that overlap with significant DMRs (promoter was defined as TSS‐5kb to TSS + 1kb). For determining the methylation status of repeats, obtained trimmed reads were mapped and methylation levels called using Bismark v0.16.3 (Krueger & Andrews, 2011) against repeat consensus sequences from repbase (Bao *et al*, 2015). Significant differentially methylated repeats were determined using logistic regression function of Seqmonk v1.47.1 (settings: five observations, all replicas, FDR < 0.05). Locations of CpG islands (CGIs) were obtained from Illingworth *et al* (2010). The Gene Expression Omnibus accession number for the BS‐Seq data reported in this paper is GSE156006.

## Author contributions

JA, RARP, and MW conceived the project and wrote the manuscript. JA and MW designed, performed, and analyzed the KD experiments, analyzed the RNA‐Seq and BS‐Seq experiments, and evaluated the results. HRC performed and analyzed RNA‐Seq experiments, evaluated the results and assisted in writing the manuscript. DM performed computational analysis of RNA‐Seq results. MPS and JS provided guidance throughout the studies, supported RNA‐Seq and BS‐Seq experiments, and assisted in writing the manuscript.

## Conflict of interest

The authors declare that they have no conflict of interest.

## Supporting information



Appendix

Expanded View Figures PDF

Dataset EV1

Dataset EV2

Dataset EV3

Dataset EV4

Dataset EV5

Movie EV1

Movie EV2

Table EV1

Table EV2

Table EV3

Table EV4

## Data Availability

The datasets produced in this study are available in the following databases: RNA‐seq data: Gene Expression Omnibus GSE57063. (https://www.ncbi.nlm.nih.gov/geo/query/acc.cgi?acc=GSE57063). Whole‐genome bisulfite‐sequencing: Gene Expression Omnibus GSE156006. (https://www.ncbi.nlm.nih.gov/geo/query/acc.cgi?acc=GSE156006).
