# Supplementary material for: Tet enzymes are essential for early embryogenesis and completion of embryonic genome activation
Source: EMBO Rep. 2021 Dec 6;23(2):e53968. doi: 10.15252/embr.202153968 (PMC8811641; doi:10.15252/embr.202153968)
Supplement: Supplementary file 1 — Appendix [file EMBR-23-e53968-s007.pdf]

Appendix for "Tet enzymes are essential for embryogenesis and completion of embryonic genome activation"

Julia Arand[1,2,3,4], H. Rosaria Chiang[2,4,5], David Martin[1], Michael P. Snyder[4], Julien Sage[2,3,4], Renee A. Reijo Pera[2,5,6], and Mark Wossidlo[1,2,4,5]*

[1]Center of Anatomy and Cell Biology, Department of Cell and Developmental Biology, Medical University of Vienna, 1090 Vienna, Austria

[2]Institute for Stem Cell Biology and Regenerative Medicine, Stanford University, Stanford, CA 94305, USA

Departments of [3]Pediatrics, [4]Genetics and [5]Obstetrics & Gynecology, Stanford University, Stanford, CA 94305, USA

[6]Current address: McLaughlin Research Institute, Great Falls, MT 59405, USA

*Correspondence to: mark.wossidlo@meduniwien.ac.at

Table of content:

Appendix Figures S1-S7

Appendix Table S1 and S2

A

Tet2 full-length (5867bp)

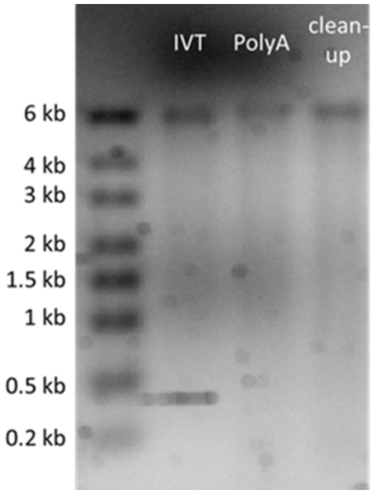

B

Tet2 truncated (3986bp)

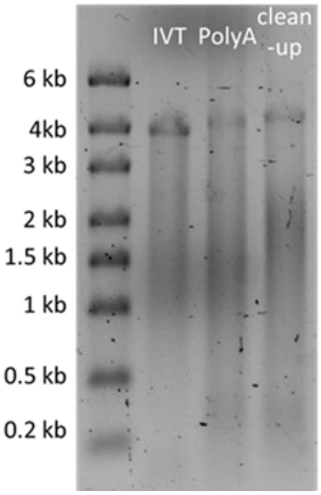

C

Template for IVT (5867 bp): Tet2 full-length (amplified from FH-Tet2-pEF-5)

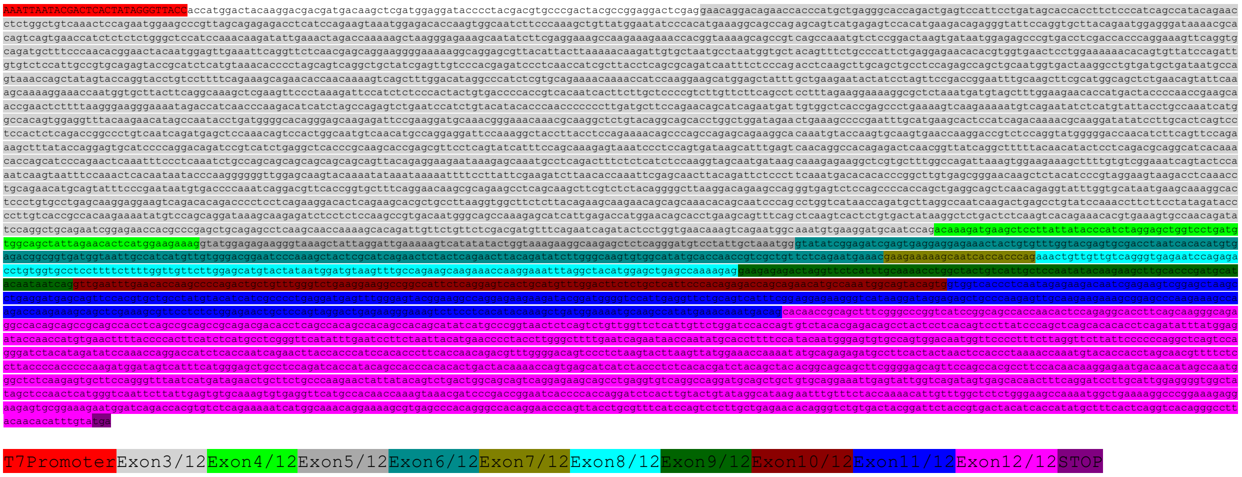

D

Template for IVT (3986 bp): Tet2 truncated (amplified from FH-Tet2-pEF-5 linearized with SpeI, mimicking Tet2 KO from Dai *et al*. Nature, 2016)

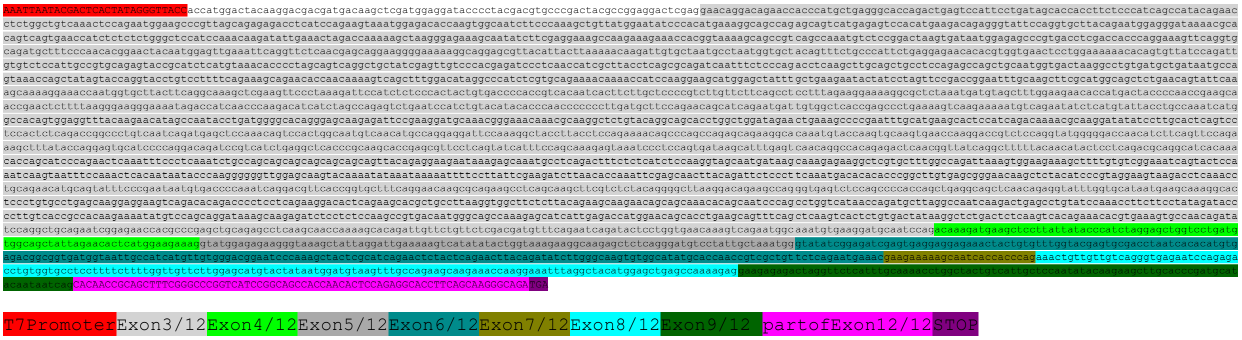

Appendix Figure S1: Generation of mRNA for Tet2 rescue experiments

(A+B) 1% denaturing agarose gel showing *in vitro* transcribed **(A)** full-length Tet2-mRNA and **(B)** truncated Tet2-mRNA used for rescue experiments after *in vitro* transcription (IVT), poly-adenylation reaction (Poly-A) and the clean-up of the reaction (clean-up).

(C+D) Sequences of generated PCR products used as template for IVT. Different exons are highlighted. **(C)** Full-length Tet2. **(D)** Truncated Tet2 without catalytical domain, mimicking the Tet2-KO from Dai *et al.,* 2016.

A

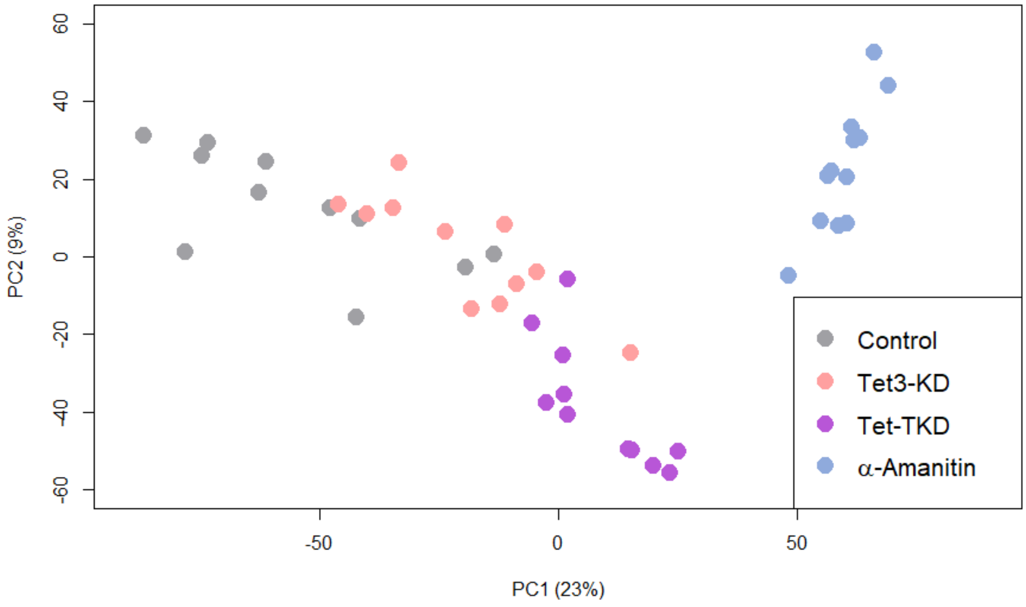

B

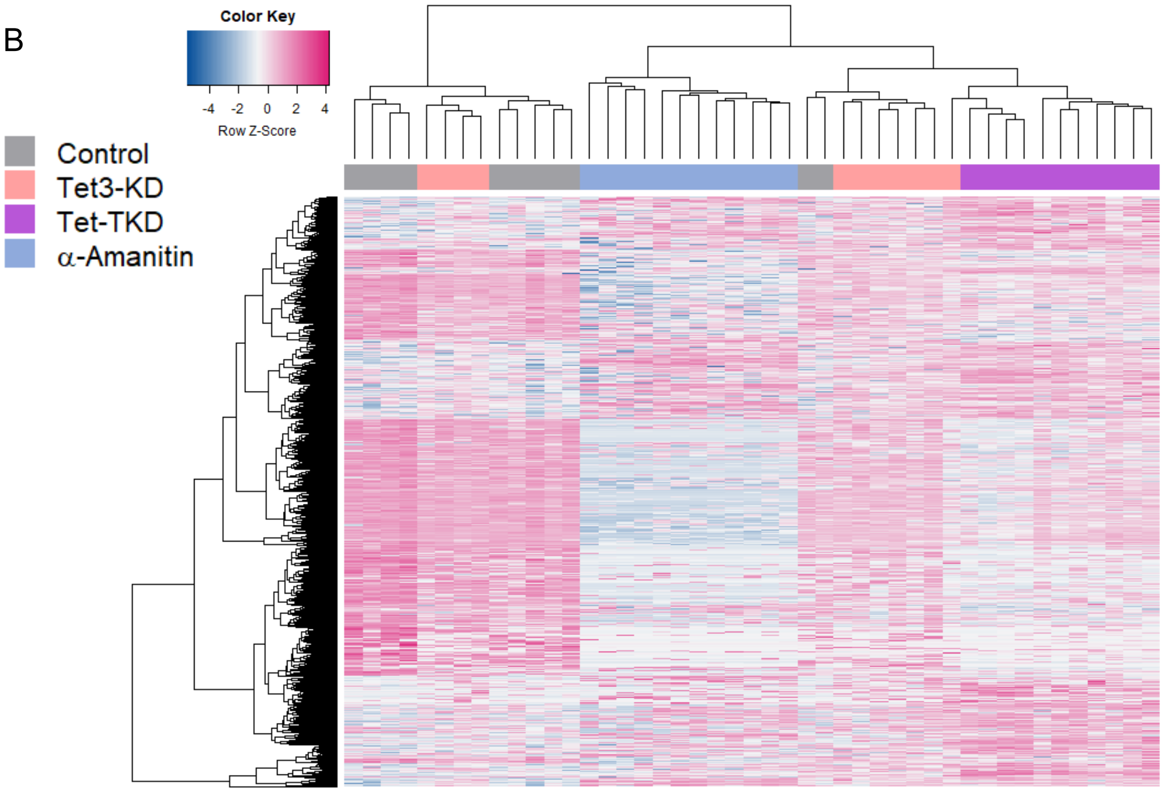

Appendix Figure S2: RNA-seq analysis of single copy genes in control, Tet3 and Tet-TKD 2-cell and α-amanitin inhibited embryos

(A) Principle component analysis of single embryo RNA-seq samples (each dot represents one 2-cell embryo).

(B) Heatmap of differentially expressed genes in derived 2-cell embryos. The map shows row-normalized expression levels for control, Tet3-KD, Tet-TKD and α-amanitin treated 2-cell embryos using unsupervised hierarchical clustering.

A

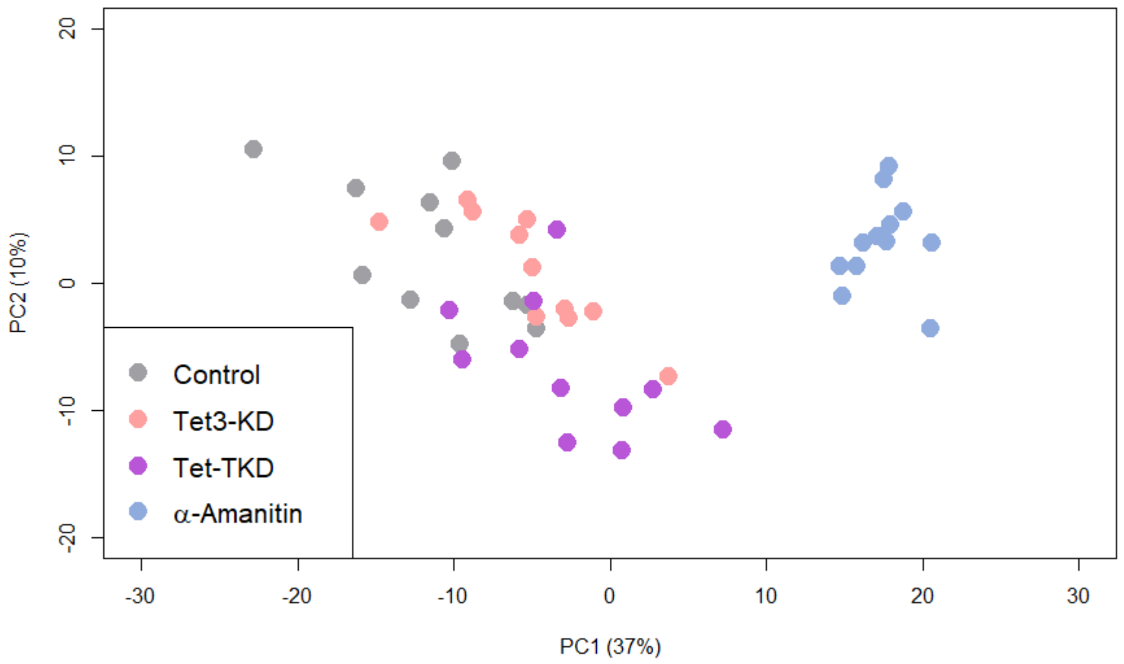

B

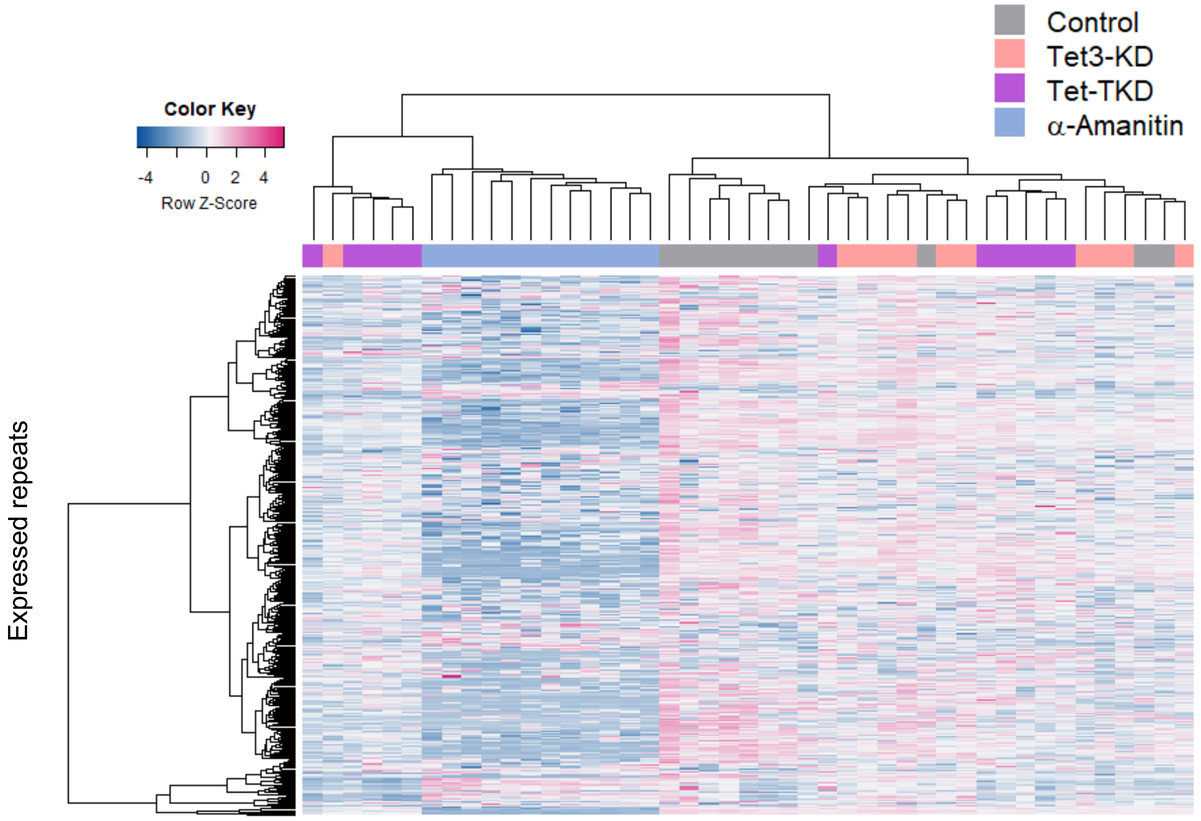

Appendix Figure S3: Expression analysis of transposable elements (TEs) in control, Tet3-KD, Tet-TKD and α-amanitin treated embryos

(A) Principle component analysis of expression levels of TEs. Each dot represents a single 2-cell embryo.

(B) Heatmap of row-normalized expression levels of TEs normalized with single genes.

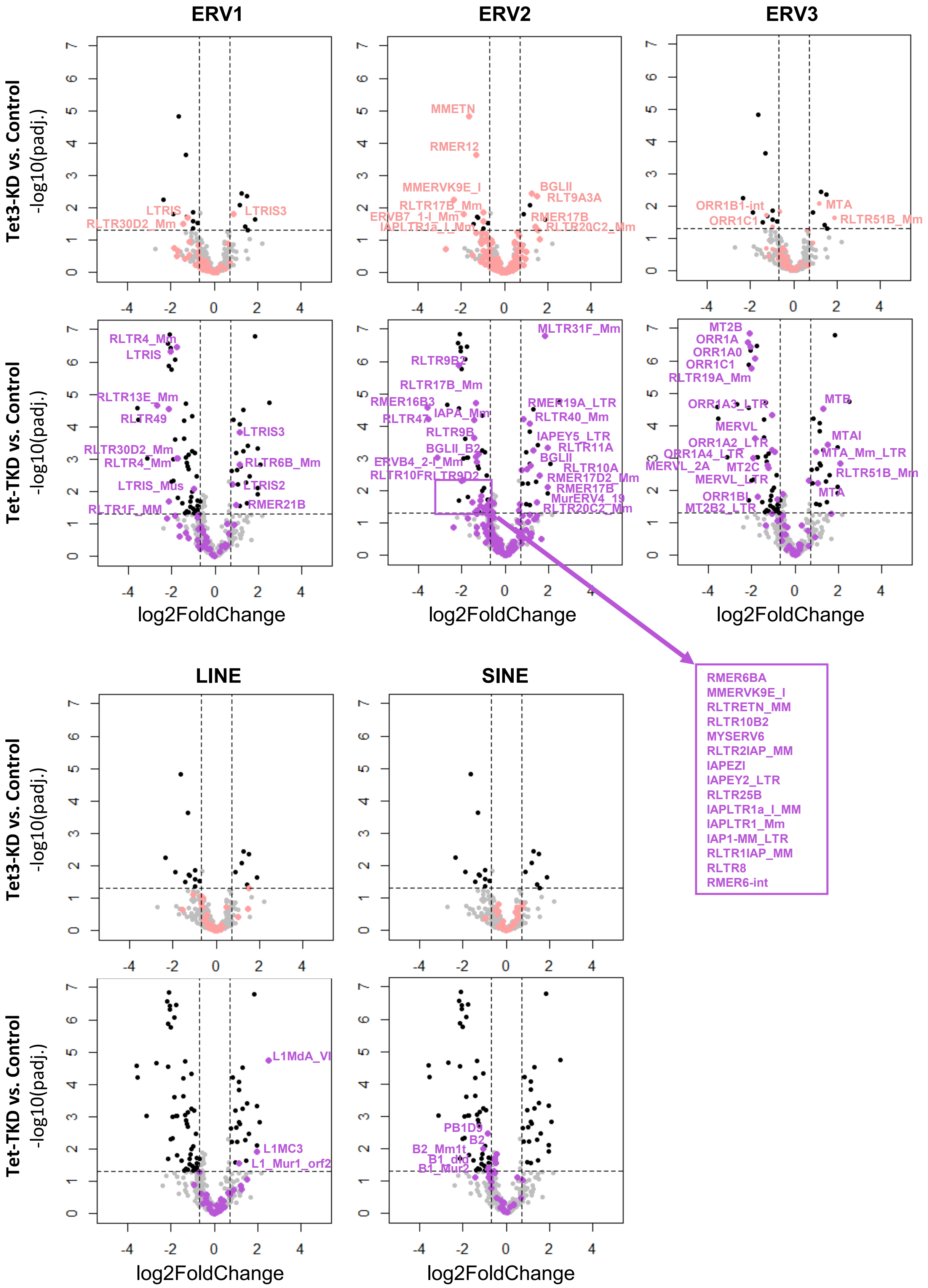

Appendix Figure S4: Differential expression of repeats in Tet3-KD and Tet-TKD 2-cell embryos compared to control 2-cell embryos.
Specific, differentially expressed repeats of the different classes are highlighted in the specific volcano plots.

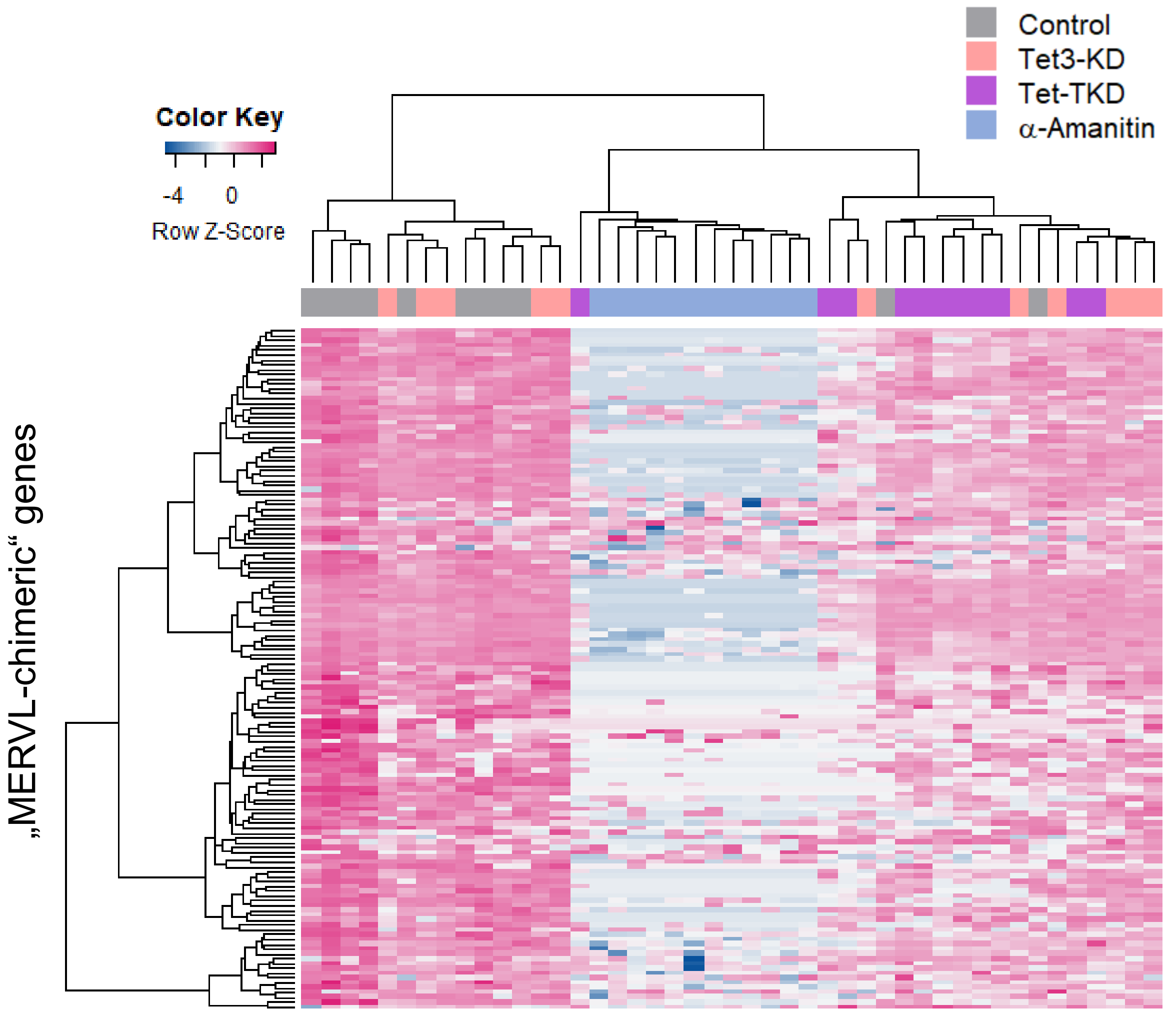

Appendix Figure S5: Hierarchical clustering of expression profiles of 2C-specific chimeric genes. Heatmap of row-normalized expression levels of genes defined by MacFarlan *et al.*, 2012 as MERVL-chimeric transcripts for control, Tet3-KD, Tet-TKD and α-amanitin treated embryos.

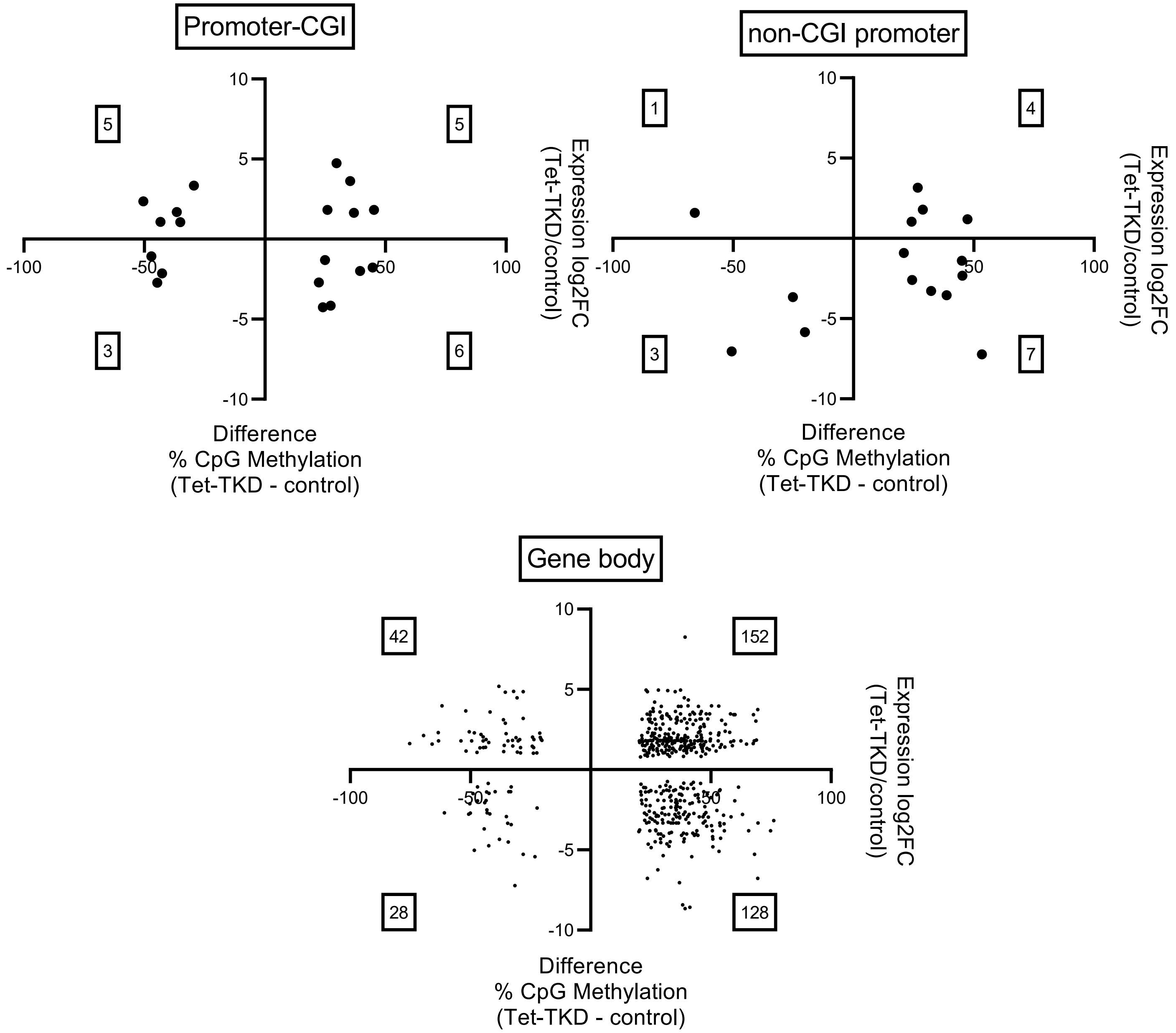

Appendix Figure S6: Correlation analysis of DNA methylation and gene expression for promoter CGIs, non-CGI promoter and gene bodies.

Shown are genes which are differentially expressed and show significant differentially methylated tiles. Number in boxes represent number of genes per quadrant.

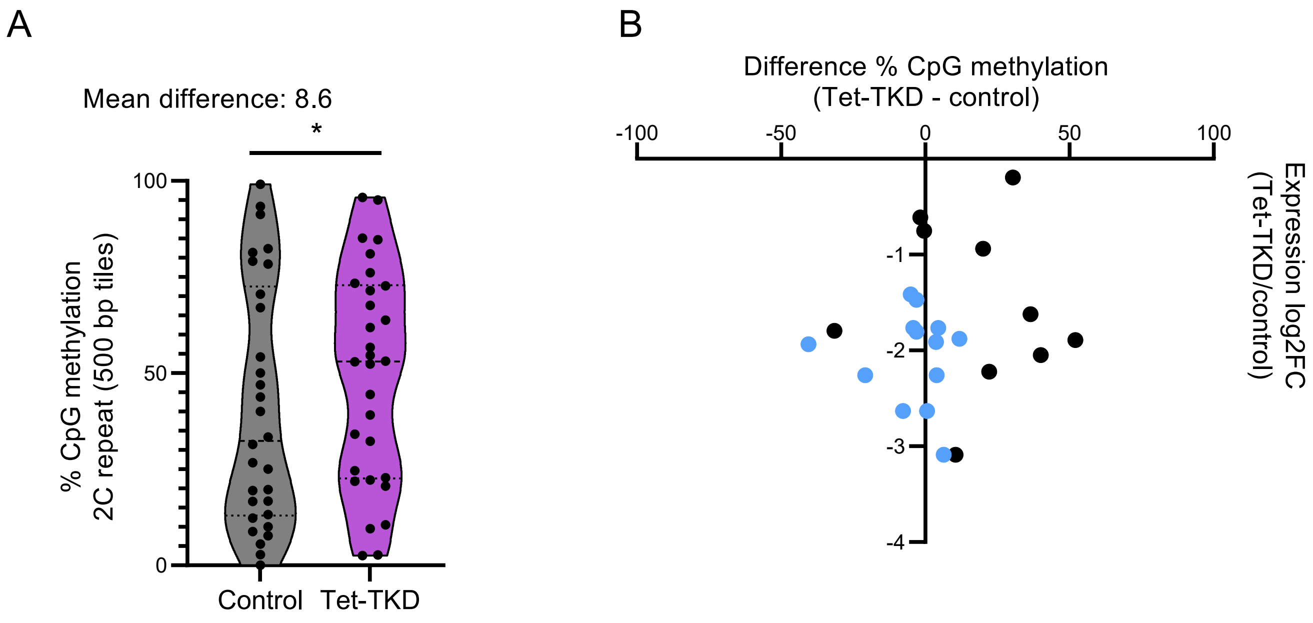

Appendix Figure S7: Correlation of DNA methylation and gene expression of MERVL-driven chimeric genes

(A) DNA Methylation of 500 bp tiles overlapping repeats of MERVL-driven chimeric genes. Data is represented as medium smoothed violin plot with indicated median and quartiles as dotted line; each dot represents one 2C repeat (two-tailed paired t-test; * = $p<0.05$). **(B)** Correlation plot of DNA methylation differences of tiles from **(A)** and corresponding gene expression. Significant differentially expressed genes are colored in blue.

Appendix Table S1: Morpholino (MO) mediated knockdown (KD) of Tet1-3 in early embryogenesis

Location of genomic binding sides for MOs targeting the 5’-UTRs of Tet1-3 mRNAs. MO-set 1 is marked in green, MO-set 2 is marked in blue. Start-codon is indicated in yellow. Tet3oo was described in Jin *et al.,* 2016.

Morpholino	Targeted 5‘-UTR
Tet1-MOs	TCCAGATTCTCCTCAAAAGGAAGATTTTGCAAAGGACCAATGACACTGTGCCCC GCATCCTTGCATCTTTACTTACTCTGCAGCCATGTCTCGGT
Tet2-MOs	GGTGTAACTGTAATGCTGTGTCTTTACTTTTTTCTGTGTCTTTAGGATTCATTC AAAGGGCAGCCTTGTGGATGGCCCCGAAGCAAGCCTGATGGAACAGGACAGAAC CACCCAT
Tet3oo- and Tet3-MOs	AGCTGCTGCTTTGGGGTCGCACATGTTCCTCCCAGAAACCCCTCAACAATATGC TGTGGAAATAAATGCTCGTGAAGGAACGGGGCCCTGGGCACAAGGGGCGACTGT CAAGACAGGCTCAGAGCTCAGCCCAGTTGATGGACCTGTTCCAGGTCAGATG
Control-MO	Mutated human beta-globin intron

Appendix Table S2: Enrichment analyses of differentially expressed genes

ChIP Enrichment Analysis of downregulated genes in Tet3-KD and Tet-TKD 2-cell embryos. List was obtained from Enrichr (Kuleshov *et al.,* 2016; Lachmann *et al.,* 2010), with an adjusted p-value of <0.1 for Tet-TKD and <0.25 for Tet3-KD applied to all samples from mouse embryonic stem cells from ChEA 2016 database, duplicated gene names were removed keeping the term with the highest adjusted p-value (padj.). Non-overlapping binding factors between both groups are marked bold (Stat3, Zfp42 and Tet1), factors that have an adjusted p-value in Tet3-KD of <0.25 & >0.1 are marked italic. PMID = Pubmed ID of study used to obtain the list of targets.

Enrichment analysis of downregulated genes to ChEA database (2016, mESCs)							
Tet3-KD				Tet-TKD			
Term	PMID	padj.	Overlap	Term	PMID	padj.	Overlap
KDM5B	21448134	1,22E-22	145/3724	KDM5B	21448134	3,18E-99	719/3724
MYC	19030024	6,65E-20	142/3868	MYC	19030024	5,59E-61	651/3868
NELFA	20434984	4,24E-14	85/2000	NELFA	20434984	5,50E-51	396/2000
ZFX	18555785	4,72E-12	110/3249	JARID1A	20064375	3,51E-40	392/2171
E2F1	18555785	2,12E-11	128/4172	E2F1	18555785	1,48E-38	621/4172
ASH2L	23239880	2,85E-09	104/3336	MYCN	18555785	1,20E-32	382/2261
JARID1A	20064375	1,98E-05	67/2171	ZFX	18555785	2,56E-32	497/3249
MYCN	18555785	1,98E-05	69/2261	KLF4	18555785	1,11E-20	365/2444
MYBL2	22936984	1,10E-04	66/2250	CHD1	19587682	5,26E-15	153/843
KLF4	18555785	2,26E-04	69/2444	ASH2L	23239880	9,22E-13	429/3336
CHD1	19587682	3,59E-03	29/843	*SIN3A*	21632747	6,96E-09	174/1186
TRIM28	19339689	4,26E-03	76/3072	*SIN3B*	21632747	5,33E-08	500/4302
CNOT3	19339689	1,80E-02	42/1547	MYBL2	22936984	6,65E-08	287/2250
TCFCP2L1	18555785	1,86E-02	51/1987	TRIM28	19339689	1,31E-06	365/3072
YY1	21170310	5,42E-02	16/464	*HCFC1*	20581084	1,35E-06	58/306
THAP11	20581084	5,82E-02	25/864	YY1	21170310	4,07E-06	77/464
NANOG	18555785	9,40E-02	17/542	THAP11	20581084	1,01E-05	123/864
SIN3B	21632747	1,37E-01	89/4302	*POU5F1*	18555785	1,52E-05	86/555
SOX2	19030024	1,55E-01	23/863	**STAT3**	18555785	4,62E-05	86/572
POU5F1	18555785	1,91E-01	16/555	*SOX2*	18555785	1,50E-04	75/497
SIN3A	21632747	1,96E-01	29/1186	**ZFP42**	18358816	1,58E-04	184/1480
HCFC1	20581084	2,32E-01	10/306	CNOT3	19339689	1,63E-04	191/1547
				NANOG	18555785	0,0012619	76/542
				TCFCP2L1	18555785	0,00167237	229/1987
				**TET1**	21451524	0,09525211	196/1839

Appendix References

Dai HQ, Wang BA, Yang L, Chen JJ, Zhu GC, Sun ML, Ge H, Wang R, Chapman DL, Tang F *et al* (2016) TET-mediated DNA demethylation controls gastrulation by regulating Lefty-Nodal signalling. *Nature* 538: 528-532

Jin SG, Zhang ZM, Dunwell TL, Harter MR, Wu X, Johnson J, Li Z, Liu J, Szabo PE, Lu Q et al (2016) Tet3 Reads 5-Carboxylcytosine through Its CXXC Domain and Is a Potential Guardian against Neurodegeneration. Cell Rep 14: 493-505

Kuleshov MV, Jones MR, Rouillard AD, Fernandez NF, Duan Q, Wang Z, Koplev S, Jenkins SL, Jagodnik KM, Lachmann A *et al* (2016) Enrichr: a comprehensive gene set enrichment analysis web server 2016 update. *Nucleic Acids Res* 44: W90-97

Lachmann A, Xu H, Krishnan J, Berger SI, Mazloom AR, Ma'ayan A (2010) ChEA: transcription factor regulation inferred from integrating genome-wide ChIP-X experiments. *Bioinformatics* 26: 2438-2444

Macfarlan TS, Gifford WD, Driscoll S, Lettieri K, Rowe HM, Bonanomi D, Firth A, Singer O, Trono D, Pfaff SL (2012) Embryonic stem cell potency fluctuates with endogenous retrovirus activity. *Nature* 487: 57-63
