# Supplementary material for: Tet enzymes are essential for early embryogenesis and completion of embryonic genome activation
Source: EMBO Rep. 2021 Dec 6;23(2):e53968. doi: 10.15252/embr.202153968 (PMC8811641; doi:10.15252/embr.202153968)
Supplement: Supplementary file 2 — Expanded View Figures PDF [file EMBR-23-e53968-s014.pdf]

Expanded View Figures

A

	Number of zygotes	Number of 2-cell embryos	Arrest at 2-cell	3-cell	4-cell	6-cell	8-cell	morula	Number of blastocyst	% blastocysts
non injected	57	50	1	0	0	0	1	11	37	74
control MO	84	79	1	0	0	1	2	9	66	83.5
control MO + Tet2 mRNA	16	14	0	0	0	0	0	1	13	92.9
Tet TKD	19	17	14	2	1	0	0	0	0	0
Tet1 KD	14	13	0	0	0	0	0	1	12	92.3
Tet2 KD	49	43	26	6	8	0	1	2	0	0
Tet3 KD	19	18	0	0	2	0	4	7	5	27.8
Tet1+2 KD	19	14	11	1	1	0	1	0	0	0
Tet1+2 KD MO2	9	7	3	0	2	1	1	0	0	0
Tet2 KD MO2	25	21	7	4	9	0	0	1	0	0
Tet2 MO2 + GFP mRNA	12	9	3	2	2	1	1	0	0	0
Tet2 MO2 + Tet2mRNA	35	30	2	1	1	4	2	12	8	26.7
Tet2 MO2 + Tet2 trunc mRNA	26	23	5	2	3	1	2	9	1	4.3

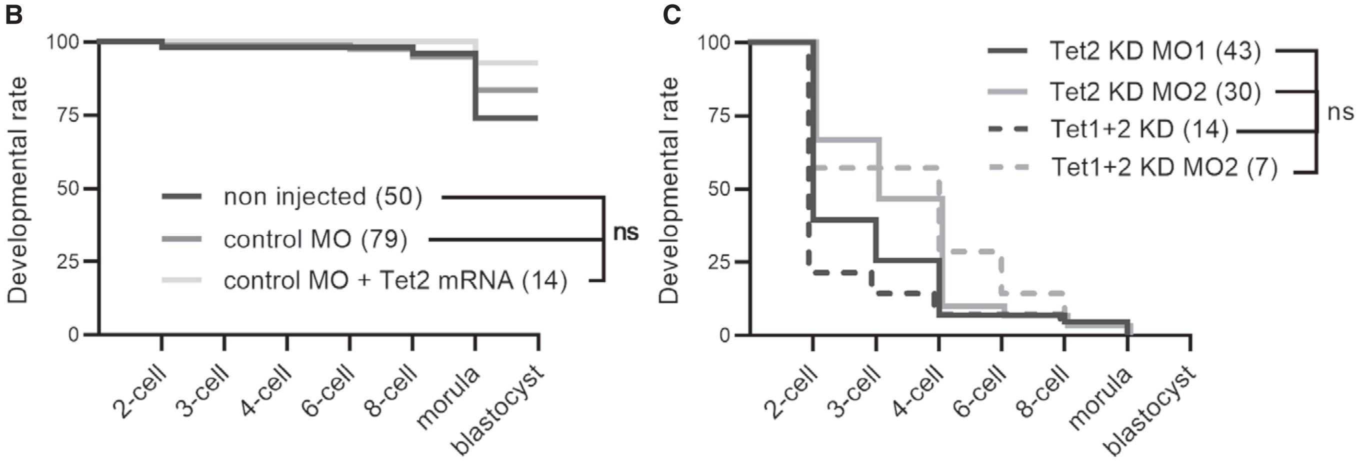

Figure EV1. Developmental rates of embryos derived from microinjected oocytes.

A Summary of all analyzed samples (% blastocysts = % 2-cell embryos developed to the blastocyst stage).

B, C Developmental curves of selected sets of experimental groups starting from 2-cell stage. (B) Comparison of control groups. (C) Comparison of Tet2-MO1 and Tet2-MO2 groups and Tet1 + 2 combined KDs. Indicated significances were tested using log rank (Mantel-Cox) test (ns = non-significant, numbers of analyzed embryos per experimental group are indicated in parenthesis).

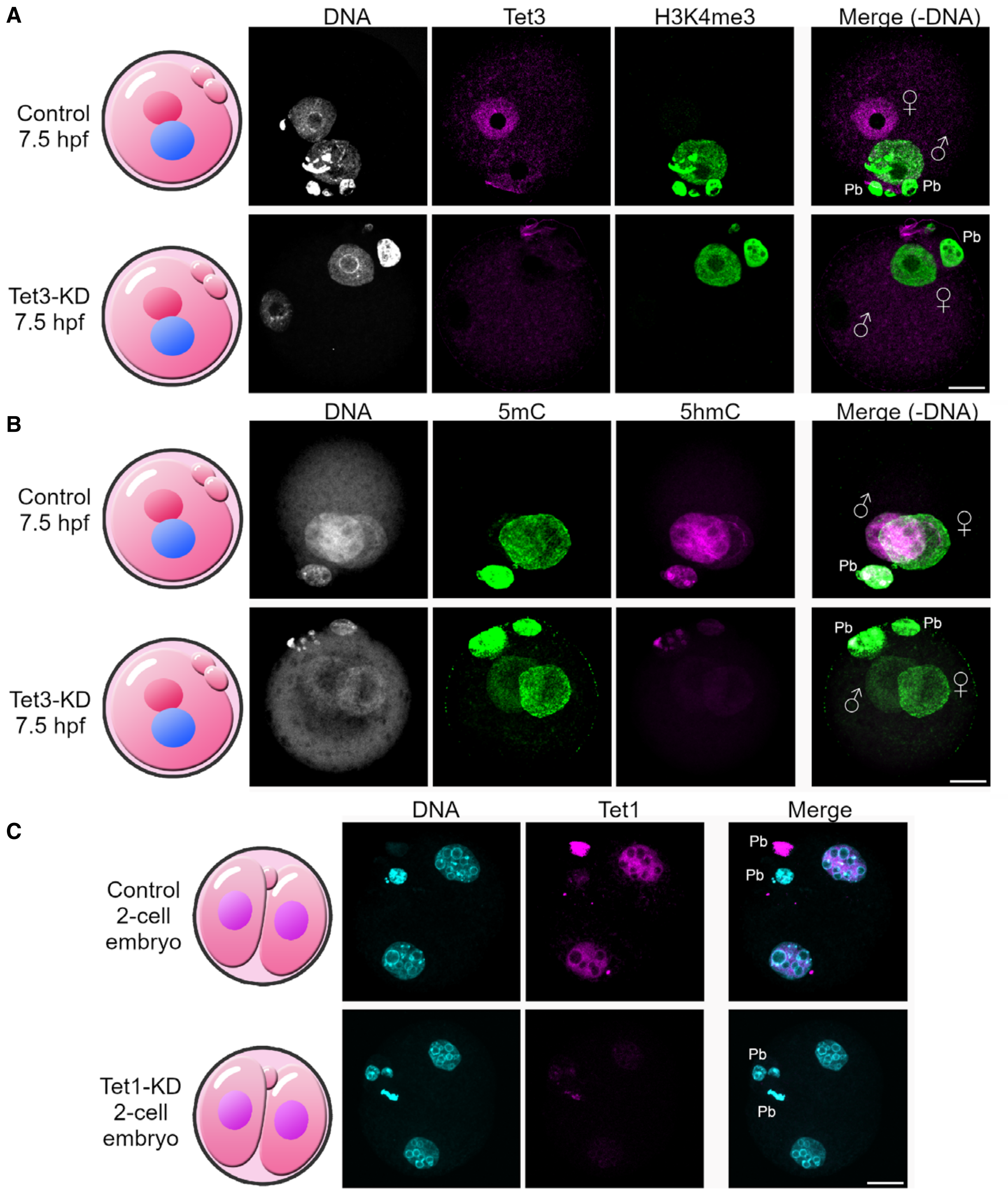

Figure EV2. Analysis of Tet3- and Tet1-knockdown efficiency.

A, B Analysis of Tet3-KD efficiency. (A) Representative images of Tet3 and H3K4me3 indirect immunofluorescence (IF) of 7.5 hpf zygotes derived from control or Tet3-KD GVOs. Tet3 can be detected in the paternal pronucleus (the maternal pronucleus is marked by H3K4me3-IF) of control derived zygotes, whereas Tet3 knockdown zygotes are negative for Tet3-signal (*n* = 12). (B) Representative images of 5mC- and 5hmC-IF of 7.5 hpf zygotes. The knockdown of Tet3 reduces the loss of 5mC and the gain of 5hmC in the paternal pronucleus (*n* = 14). Paternal and maternal pronuclei are indicated.

C Analysis of Tet1-KD efficiency. Representative images of Tet1 IF of G2-phase 2-cell embryos (32 hpf) of control or Tet1-KD-derived GVOs (*n* = 12). Control embryos show nuclear Tet1 signal, whereas Tet1-KD embryos show greatly reduced Tet1 signal.

Data information: Paternal and maternal pronuclei are indicated, Pb = polar body, scale bar = 20 μm.

 © 2021 The Authors

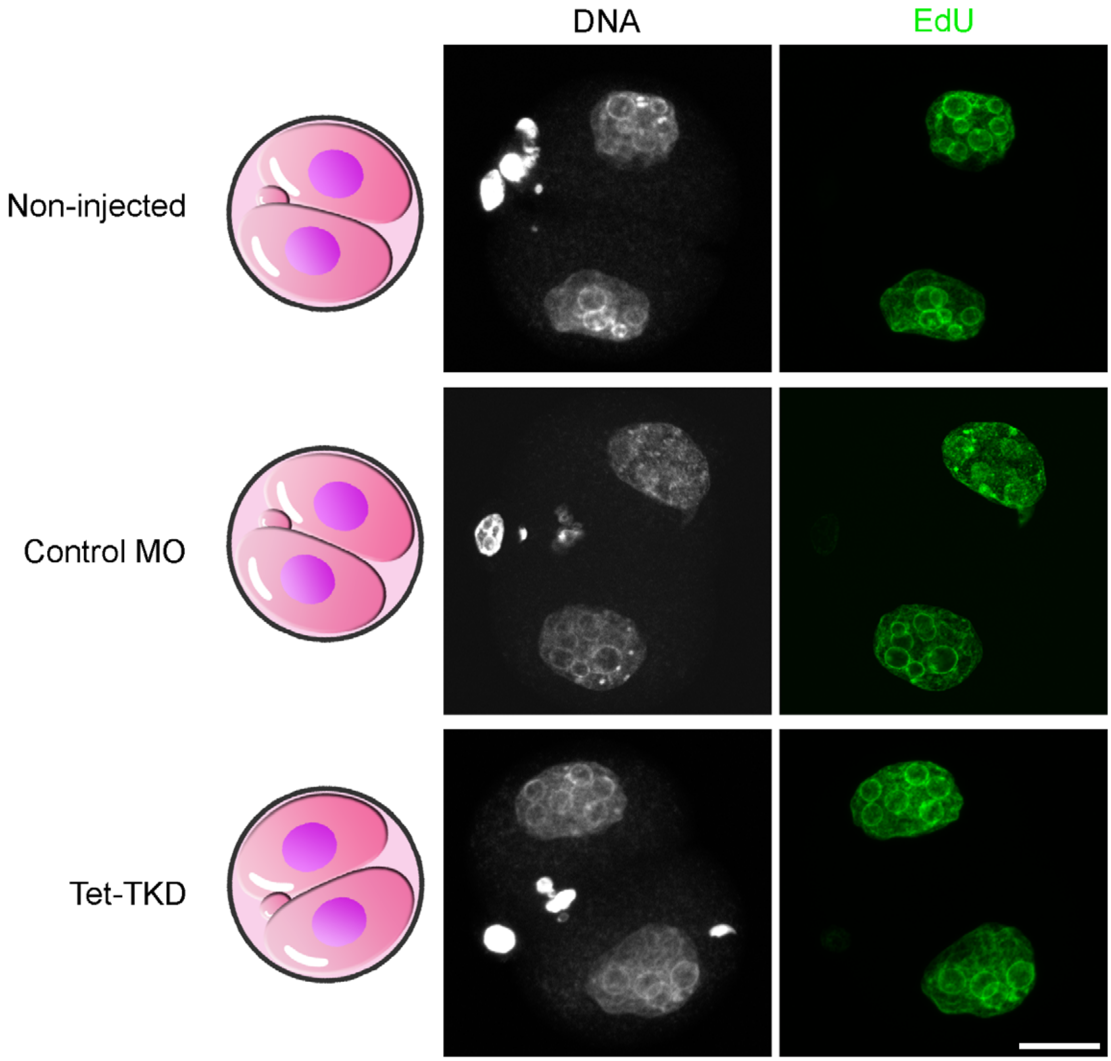

Figure EV3. Tet-TKD embryos undergo S-phase in the 2-cell stage.

Representative images of 2-cell embryos derived from non-injected, control-morpholino, or Tet-1–3 MO injected GVOs, which were incubated with EdU from 22.5 hpf until 27.5 hpf and analyzed for EdU incorporation. All three groups show similar incorporation of EdU indicating that Tet-TKD 2-cell embryos undergo replication in the 2-cell stage (Tet-TKD: $n = 5$, non-injected: $n = 3$, control-MO: $n = 2$; scale bar = 20 μm).

© 2021 The Authors

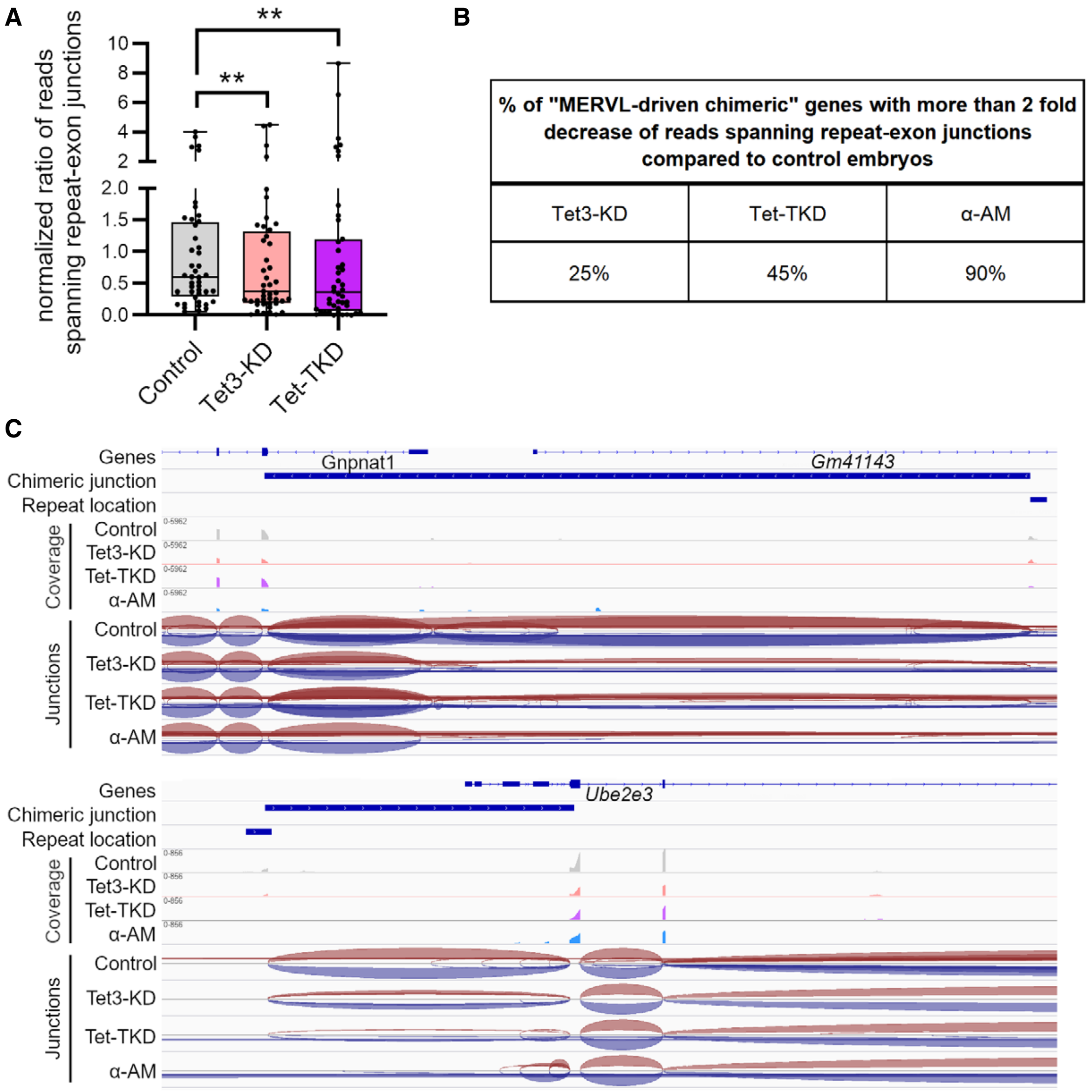

Figure EV4. Expression analysis of MERVL-driven chimeric transcripts.

A Normalized ratio of reads spanning the repeat-exon junctions of "MERVL-chimeric" genes according to MacFarlan *et al* (2012). Statistical significance was tested using Friedman test with Dunn's multiple comparisons test (**$P < 0.01$). Data from all embryos of one condition (11 embryos each) were merged for this analysis. Data are represented as box plots with indicated mean and quartiles with whiskers representing the min and max values; dots represent different repeat-exon junctions.

B Percent of analyzed "MERVL-driven chimeric" genes, which show a 2-fold decrease of the usage of the chimeric junction compared to control embryos. RNA-seq data from all embryos of one condition were merged for this analysis. Note: In α-Amatinin (α-AM) 2-cell embryos for 85% of genes no junctions could be observed (only genes which had more than 1,000 reads mapping to the whole transcript were considered).

C Screenshots of IGV genome browser showing RNA-seq data of control, Tet3-KD, Tet-TKD, and α-AM treated 2-cell embryos at chimeric repeat-exon junction sites of the "2C-genes" Gnpnat1 and Ube2e3.

 © 2021 The Authors

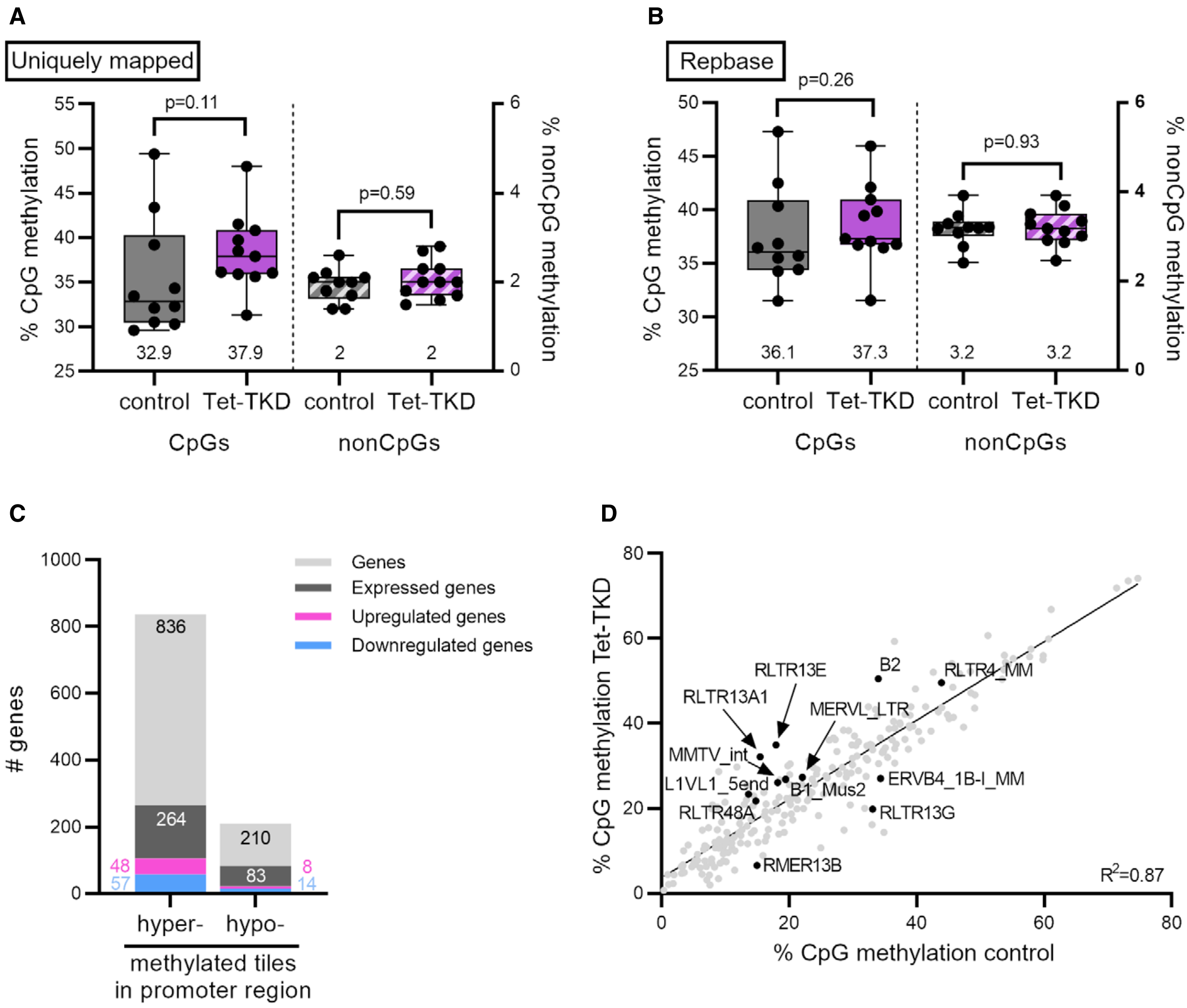

Figure EV5. BS-Seq analysis of control and Tet-TKD 2-cell embryos.

A, B Overall methylation levels of cytosines in CpG context and non-CpG context in (A) uniquely mapped reads or (B) reads mapping to reference sequences obtained from Repbase (Bao *et al*, 2015). Numbers indicated below the box plot represent the median. Data is represented as box plots, with indicated median and quartiles with whiskers representing the min and max values; dots represent single 2-cell embryos (control n = 10, Tet-TKD n = 11). Statistical significance was tested using Mann–Whitney test.

C Number of genes associated with hypo- or hypermethylated 500-bp tiles in their promoter region (TSS −5 kb/+1 kb).

D Correlation of CpG methylation of repetitive elements in control and Tet-TKD 2-cell embryos. Indicated are significantly differentially methylated genes with a methylation difference of > 5% and an adjusted P-value < 0.05. % CpG methylation corresponds to the methylation levels in the merged datasets of each condition over the annotated repeat element.

© 2021 The Authors
