# Supplementary material for: Tet enzymes are essential for early embryogenesis and completion of embryonic genome activation
Source: EMBO Rep. 2021 Dec 6;23(2):e53968. doi: 10.15252/embr.202153968 (PMC8811641; doi:10.15252/embr.202153968)
Supplement: Supplementary file 8 — Movie EV1 [file EMBR-23-e53968-s003.zip › Movie_EV1-legend.docx]

**Movie EV1** – Time-lapse movie of Tet-TKD (columns A+B) and control derived embryos (columns C+D) from 7 hpf to 3.5 dpf, images were taken every 5 min and compiled in a movie with 20 fps.
