# Supplementary material for: Tet enzymes are essential for early embryogenesis and completion of embryonic genome activation
Source: EMBO Rep. 2021 Dec 6;23(2):e53968. doi: 10.15252/embr.202153968 (PMC8811641; doi:10.15252/embr.202153968)
Supplement: Supplementary file 14 [file EMBR-23-e53968-s008.pdf]

EMBO reports

Tet enzymes are essential for early embryogenesis and completion of embryonic genome activation

Julia Arand, H. Rosaria Chiang, David Martin, Michael Snyder, Julien Sage, Renee Reijo Pera, and Mark Wossidlo
DOI: 10.15252/embr.202153968

Corresponding author(s): Mark Wossidlo (mark.wossidlo@meduniwien.ac.at)

Review Timeline:

Submission Date:	9th Sep 21
Editorial Decision:	4th Oct 21
Revision Received:	20th Oct 21
Editorial Decision:	15th Nov 21
Revision Received:	17th Nov 21
Accepted:	18th Nov 21

Editor: Martina Rembold

Transaction Report:

Please note that the manuscript was previously reviewed at another journal and the reports were taken into account in the decision making process at EMBO reports. Since the original reviews are not subject to EMBO Press' transparent review process policy, the reports and author response cannot be published.

1st Editorial Decision 4th Oct 2021

Dear Mark,

Thank you for the submission of your research manuscript to our journal. You report that Tet enzymes are essential for early mouse development and genome activation. Your study had been reviewed and revised for another journal that could not offer publication. We note that referee 1 supported publication after revision, while referee 2 remained concerned that the study remained descriptive without offering mechanistic insight into the catalytic and non-catalytic roles of TET2 in early development and gene expression. Referee 3 remained concerned regarding the use of a truncated form of Tet2 instead of a catalytically inactive full-length Tet2 in the rescue experiments. We also note that referee 1 and 3 considered the finding that Tet enzymes play a role in genome activation and early mouse development of interest to the community and given that EMBO reports does not require a full mechanistic understanding, we considered the study a potentially good fit for publication in EMBO reports.

We have therefore discussed the remaining concerns further with an expert in the field. The expert agreed that the data on the Tet2 truncation construct should remain part of the study but that it needs to be complemented with an extended discussion of the value of the chosen truncation construct and potential alternative scenarios and limitations, such as the disruption of protein-protein interactions. Furthermore, we asked the advisor to comment on the specificity of the morpholino-based knockdown approach, which was one of the concerns raised during review. The expert advisor noted also that the phenotype you observe upon MO-induced knockdown of Tet enzymes in oocytes differs from that reported for germline-deleted Tet2 mice, which are fertile. However, the advisor also acknowledged the different approaches and supported publication, if the contradiction between the fecundity of germline-deleted Tet2 KO mice and your observations with morpholino-injected oocytes are explicitly acknowledged and thoroughly discussed.

Based on this evaluation and the advisor's feedback, we have decided to proceed with the publication of your manuscript, pending that the above-mentioned issues are appropriately discussed.

In addition, please format your manuscript according to the following guidelines below. Please note that we will perform a quality control on the revised manuscript, that involves a check of all figures and their legends. This step is usually done on the revised manuscript before re-review but in this case, it will have to be done before acceptance. In order to make this process smooth, please carefully review the instructions below.

Before listing general guidelines, I list a few items specific to your manuscript:

- Your manuscript has 5 figures and will therefore be published in our Reports section. To do so, please combine the Results and Discussion sections and please keep an eye on our character count of 25,000 (plus/minus 2,000) for the main text. If you need more space for the discussion, please let me know so we can discuss the layout again.

- Please rewrite the abstract in present tense and shorten it to 175 words. I also suggest to mention the morpholino-based approach in the abstract, as it currently seems to imply the analysis of KO embryos.

- Supplementary figure S12: Please define the central band, boxes and whiskers of the boxplot.

Please check all figure legends and make sure that the following information is present:
- the name of the statistical test used to generate error bars and P values,
- the number (n) of independent experiments (please specify technical or biological replicates) underlying each data point,
- the nature of the bars and error bars (s.d., s.e.m.)
- If the data are obtained from n {less than or equal to} 2, use scatter blots showing the individual data points and do not calculate statistics.

Discussion of statistical methodology can be reported in the materials and methods section, but figure legends should contain a basic description of n, P and the test applied.
See also the guidelines for figure legend preparation:
https://www.embopress.org/page/journal/14693178/authorguide#figureformat
- Please also include scale bars in all microscopy images.

When submitting your revised manuscript, we will require:

1) a .docx formatted version of the manuscript text (including legends for main figures, EV figures and tables). Please make sure that the changes are highlighted to be clearly visible.

2) individual production quality figure files as .eps, .tif, .jpg (one file per figure).
Please download our Figure Preparation Guidelines (figure preparation pdf) from our Author Guidelines pages https://www.embopress.org/page/journal/14693178/authorguide for more info on how to prepare your figures.

3) a .docx formatted letter INCLUDING the reviewers' reports and your detailed point-by-point responses to their comments. As part of the EMBO Press transparent editorial process, the point-by-point response is part of the Review Process File (RPF), which will be published alongside your paper.

4) a complete author checklist, which you can download from our author guidelines (<https://www.embopress.org/page/journal/14693178/authorguide>). Please insert information in the checklist that is also reflected in the manuscript. The completed author checklist will also be part of the RPF.

5) Please note that all corresponding authors are required to supply an ORCID ID for their name upon submission of a revised manuscript (<https://orcid.org/>). Please find instructions on how to link your ORCID ID to your account in our manuscript tracking system in our Author guidelines (<https://www.embopress.org/page/journal/14693178/authorguide#authorshipguidelines>)

6) We replaced Supplementary Information with Expanded View (EV) Figures and Tables that are collapsible/expandable online. A maximum of 5 EV Figures can be typeset. EV Figures should be cited as 'Figure EV1, Figure EV2" etc... in the text and their respective legends should be included in the main text after the legends of regular figures.

- For the figures that you do NOT wish to display as Expanded View figures, they should be bundled together with their legends in a single PDF file called *Appendix*, which should start with a short Table of Content. Appendix figures should be referred to in the main text as: "Appendix Figure S1, Appendix Figure S2" etc. See detailed instructions regarding expanded view here: <https://www.embopress.org/page/journal/14693178/authorguide#expandedview>

- Additional Tables/Datasets should be labeled and referred to as Table EV1, Dataset EV1, etc. Legends have to be provided in a separate tab in case of .xls files. Alternatively, the legend can be supplied as a separate text file (README) and zipped together with the Table/Dataset file.

7) Before submitting your revision, primary datasets (and computer code, where appropriate) produced in this study need to be deposited in an appropriate public database (see < https://www.embopress.org/page/journal/14693178/authorguide#dataavailability>).
<optional: Specifically, we would kindly ask you to provide public access to the following datasets:
- RNA-seq
- Whole genome bisulfite-sequencing

Please remember to provide a reviewer password if the datasets are not yet public.

The accession numbers and database should be listed in a formal "Data Availability " section (placed after Materials & Method) that follows the model below (see also < https://www.embopress.org/page/journal/14693178/authorguide#dataavailability>). Please note that the Data Availability Section is restricted to new primary data that are part of this study.

Data availability

The datasets (and computer code) produced in this study are available in the following databases:

- RNA-Seq data: Gene Expression Omnibus GSE46843 (https://www.ncbi.nlm.nih.gov/geo/query/acc.cgi?acc=GSE46843)
- [data type]: [name of the resource] [accession number/identifier/doi] ([URL or identifiers.org/DATABASE:ACCESSION])

*** Note - All links should resolve to a page where the data can be accessed. ***

8) We would also encourage you to include the source data for figure panels that show essential data. Numerical data should be provided as individual .xls or .csv files (including a tab describing the data). For blots or microscopy, uncropped images should be submitted (using a zip archive if multiple images need to be supplied for one panel). Additional information on source data and instruction on how to label the files are available <https://www.embopress.org/page/journal/14693178/authorguide#sourcedata>.

9) Our journal encourages inclusion of *data citations in the reference list* to directly cite datasets that were re-used and obtained from public databases. Data citations in the article text are distinct from normal bibliographical citations and should directly link to the database records from which the data can be accessed. In the main text, data citations are formatted as follows: "Data ref: Smith et al, 2001" or "Data ref: NCBI Sequence Read Archive PRJNA342805, 2017". In the Reference list, data citations must be labeled with "[DATASET]". A data reference must provide the database name, accession number/identifiers and a resolvable link to the landing page from which the data can be accessed at the end of the reference. Further instructions are available at <https://www.embopress.org/page/journal/14693178/authorguide#referencesformat>.

10) As part of the EMBO publication's Transparent Editorial Process, EMBO reports publishes online a Review Process File to

accompany accepted manuscripts. This File will be published in conjunction with your paper and will include the referee reports, your point-by-point response and all pertinent correspondence relating to the manuscript.

You are able to opt out of this by letting the editorial office know (emboreports@embo.org). If you do opt out, the Review Process File link will point to the following statement: "No Review Process File is available with this article, as the authors have chosen not to make the review process public in this case."

11) Finally, EMBO reports papers are accompanied online by A) a short (1-2 sentences) summary of the findings and their significance, B) 2-3 bullet points highlighting key results and C) a synopsis image that is 550x200-600 pixels large (width x height) in .png format. You can either show a model or key data in the synopsis image. Please note that the size is rather small and that text needs to be readable at the final size. Please send us this information along with the revised manuscript.

We look forward to seeing a final version of your manuscript as soon as possible.

Kind regards,
Martina

Martina Rembold, PhD
Senior Editor
EMBO reports

Dear Mark,

Thank you for the submission of your research manuscript to our journal. You report that Tet enzymes are essential for early mouse development and genome activation. Your study had been reviewed and revised for another journal that could not offer publication. We note that referee 1 supported publication after revision, while referee 2 remained concerned that the study remained descriptive without offering mechanistic insight into the catalytic and non-catalytic roles of TET2 in early development and gene expression. Referee 3 remained concerned regarding the use of a truncated form of Tet2 instead of a catalytically inactive full-length Tet2 in the rescue experiments. We also note that referee 1 and 3 considered the finding that Tet enzymes play a role in genome activation and early mouse development of interest to the community and given that EMBO reports does not require a full mechanistic understanding, we considered the study a potentially good fit for publication in EMBO reports.

We have therefore discussed the remaining concerns further with an expert in the field. The expert agreed that the data on the Tet2 truncation construct should remain part of the study but that it needs to be complemented with an extended discussion of the value of the chosen truncation construct and potential alternative scenarios and limitations, such as the disruption of protein-protein interactions. Furthermore, we asked the advisor to comment on the specificity of the morpholino-based knockdown approach, which was one of the concerns raised during review. The expert advisor noted also that the phenotype you observe upon MO-induced knockdown of Tet enzymes in oocytes differs from that reported for germline-deleted Tet2 mice, which are fertile. However, the advisor also acknowledged the different approaches and supported publication, if the contradiction between the fecundity of germline-deleted Tet2 KO mice and your observations with morpholino-injected oocytes are explicitly acknowledged and thoroughly discussed.

Based on this evaluation and the advisor's feedback, we have decided to proceed with the publication of your manuscript, pending that the above-mentioned issues are appropriately discussed.

We thank the expert for his fair assessment of our work. We now acknowledge and discuss the contradiction of our morpholino-based model to the KO model in greater detail in several parts of our revised manuscript and include an extended discussion of the truncated Tet2 construct applied in our study. Here is a summary of the parts discussing these aspects:

Page 4: “Observed Tet-KO phenotypes are obvious during postimplantation development, with preimplantation embryos showing mild transcriptomic changes or developmental impairment in different contexts. Here, heterozygous Tet3-KO mice displayed neonatal abnormalities, and Tet1-/- and Tet2-/- single KO-embryos developed with postnatal malignancies (Dawlaty et al, 2011; Gu et al., 2011; Ko et al, 2011; Li et al, 2011).”

Page 6: “Our observation using a morpholino-based knockdown approach in fully grown oocytes stands in contrast to phenotypes observed upon germline deletion of all three Tet-enzymes, where phenotypes are emerging in early postimplantation development (Dai et al., 2016). Both approaches have advantages and limitations and target different questions. The Tet-TKO approach diminishes Tet

proteins during the growth phase of the oocyte – a phase where massive DNA remethylation is still occurring and compensation mechanisms can intervene, while the morpholino-based knockdown approach is effective in fully grown and transcriptionally silent oocytes, minimizing compensatory effects and only mimicking loss of Tet function specifically during the oocyte-to-embryo transition. Moreover, the Tet-TKO and most single Tet-KO models aimed to create genomic deletions of the catalytical domain of Tet enzymes leaving the expression of a truncated product (Dai et al., 2016; Gu et al., 2011; Ko et al., 2011; Zhang et al, 2013), while the morpholino-based KD diminishes the expression of the complete gene product. Albeit Cre/lox mediated KO approaches are highly specific and KD approaches bare possibilities of unspecific off-target effects, KO strategies can lead to unspecific phenotypes due to non-sense mediated decay of truncation constructs (El-Brolosy et al., 2019; Ma et al., 2019; Wilkinson, 2019), whereas in MO-based KD approaches the full-length RNA is still available but blocked for translation.

Page 8: "These observations for Tet2-KD were unexpected and are in contrast to published Tet2-KO models, similarly to the Tet-TKD/Tet-TKO discrepancy (Ko et al., 2011; Li et al., 2011)."

Page 9: "Thus, our rescue experiments validated the specific phenotype we observed in Tet2-KD embryos, which differs from the reported phenotype in Tet2-KO mice, which develop to term and are fertile (Ko et al., 2011; Li et al., 2011).

In summary, our analysis of Tet-KD experiments revealed a striking early developmental arrest of Tet-TKD embryos primarily at the 2-cell stage upon acute depletion of Tet enzymes in the oocyte-to-embryo transition, with the most severe phenotype linked to Tet2."

Page 18: "We designed a rescue experiment for Tet2 using a truncated Tet2 mRNA mimicking the catalytical KO of Tet2 in previous germline Tet-TKO study (Dai et al., 2016)(Appendix Fig. 4B and D). While truncated expression products of genes can mediate non-sense mediated decay, as discussed as a complication in KO approaches (Wilkinson, Nature 2019), or lead to disruptions of protein-protein interactions, a shorter isoform of Tet2 (missing the catalytical domain) is specifically expressed in human oocytes and early human cleavage stage embryos, suggesting an important non-catalytic function of the C-terminal part of Tet2 (Hendrickson et al., 2017)."

In addition, please format your manuscript according to the following guidelines below. Please note that we will perform a quality control on the revised manuscript, that involves a check of all figures and their legends. This step is usually done on the revised manuscript before re-review but in this case, it will have to be done before acceptance. In order to make this process smooth, please carefully review the instructions below.

Before listing general guidelines, I list a few items specific to your manuscript:

- Your manuscript has 5 figures and will therefore be published in our Reports section. To do so, please combine the Results and Discussion sections and please keep an eye on our character count of 25,000 (plus/minus 2,000) for the main text. If you need more space for the discussion, please let me know so we can discuss the layout again.

We reformatted the text to fit to the "report" style (combined "Results and Discussion")

- Please rewrite the abstract in present tense and shorten it to 175 words. I also suggest to mention the morpholino-based approach in the abstract, as it currently seems to imply the analysis of KO embryos.

We shortened, rewrote in present tense, and now mention the morpholino-based approach and thank you for the suggestion.

- Supplementary figure S12: Please define the central band, boxes and whiskers of the boxplot.

We now define this in the figure legend.

Please check all figure legends and make sure that the following information is present:
- the name of the statistical test used to generate error bars and P values,
- the number (n) of independent experiments (please specify technical or biological replicates) underlying each data point,
- the nature of the bars and error bars (s.d., s.e.m.)
- If the data are obtained from n {less than or equal to} 2, use scatter blots showing the individual data points and do not calculate statistics.

Discussion of statistical methodology can be reported in the materials and methods section, but figure legends should contain a basic description of n, P and the test applied.
See also the guidelines for figure legend preparation:
https://www.embopress.org/page/journal/14693178/authorguide#figureformat
- Please also include scale bars in all microscopy images.

We checked all figures legends and added the missing information and added the missing scalebars to the figures where needed, we also moved five figures from the Appendix to EV figures.

When submitting your revised manuscript, we will require:

1) a .docx formatted version of the manuscript text (including legends for main figures, EV figures and tables). Please make sure that the changes are highlighted to be clearly visible.

2) individual production quality figure files as .eps, .tif, .jpg (one file per figure).
Please download our Figure Preparation Guidelines (figure preparation pdf) from our Author Guidelines pages
https://www.embopress.org/page/journal/14693178/authorguide for more info on how to prepare your figures.

3) a .docx formatted letter INCLUDING the reviewers' reports and your detailed point-by-point responses to their comments. As part of the EMBO Press transparent editorial process, the point-by-point response is part of the Review Process File (RPF), which will be published alongside your paper.

4) a complete author checklist, which you can download from our author guidelines (<https://www.embopress.org/page/journal/14693178/authorguide>;). Please insert information in the checklist that is also reflected in the manuscript. The completed author checklist will also be part of the RPF.

5) Please note that all corresponding authors are required to supply an ORCID ID for their name upon submission of a revised manuscript (<https://orcid.org/>;). Please find instructions on how to link your ORCID ID to your account in our manuscript tracking system in our Author guidelines (<https://www.embopress.org/page/journal/14693178/authorguide#authorshipguidelines>;)

6) We replaced Supplementary Information with Expanded View (EV) Figures and Tables that are collapsible/expandable online. A maximum of 5 EV Figures can be typeset. EV Figures should be cited as 'Figure EV1, Figure EV2" etc... in the text and their respective legends should be included in the main text after the legends of regular figures.

- For the figures that you do NOT wish to display as Expanded View figures, they should be bundled together with their legends in a single PDF file called *Appendix*, which should start with a short Table of Content. Appendix figures should be referred to in the main text as: "Appendix Figure S1, Appendix Figure S2" etc. See detailed instructions regarding expanded view here: <https://www.embopress.org/page/journal/14693178/authorguide#expandedview>;

- Additional Tables/Datasets should be labeled and referred to as Table EV1, Dataset EV1, etc. Legends have to be provided in a separate tab in case of .xls files. Alternatively, the legend can be supplied as a separate text file (README) and zipped together with the Table/Dataset file.

We moved 5 figures from our Appendix into EV figures and uploaded individual figure files accordingly.

7) Before submitting your revision, primary datasets (and computer code, where appropriate) produced in this study need to be deposited in an appropriate public database (see < https://www.embopress.org/page/journal/14693178/authorguide#dataavailability>;).
<optional: Specifically, we would kindly ask you to provide public access to the following datasets:
- RNA-seq
- Whole genome bisulfite-sequencing

Please remember to provide a reviewer password if the datasets are not yet public.

The accession numbers and database should be listed in a formal "Data Availability " section (placed after Materials & Method) that follows the model below (see also < https://www.embopress.org/page/journal/14693178/authorguide#dataavailability>;). Please note that the Data Availability Section is restricted to new primary data that are part of this study.

Data availability

The datasets (and computer code) produced in this study are available in the following databases:

- RNA-Seq data: Gene Expression Omnibus GSE46843 (https://www.ncbi.nlm.nih.gov/geo/query/acc.cgi?acc=GSE46843)
- [data type]: [name of the resource] [accession number/identifier/doi] ([URL or identifiers.org/DATABASE:ACCESSION])

*** Note - All links should resolve to a page where the data can be accessed. ***

Our generated RNA-Seq and BS-Seq datasets are uploaded on Gene Expression Omnibus and will be made directly publicly available upon acceptance of our manuscript. So far the datasets can be accessed using following reviewer passwords:

reviewer passwords removed

8) We would also encourage you to include the source data for figure panels that show essential data. Numerical data should be provided as individual .xls or .csv files (including a tab describing the data). For blots or microscopy, uncropped images should be submitted (using a zip archive if multiple images need to be supplied for one panel). Additional information on source data and instruction on how to label the files are available <https://www.embopress.org/page/journal/14693178/authorguide#sourcedata>;.

We provide essential data in the Tables EV1-9.

9) Our journal encourages inclusion of *data citations in the reference list* to directly cite datasets that were re-used and obtained from public databases. Data citations in the article text are distinct from normal bibliographical citations and should directly link to the database records from which the data can be accessed. In the main text, data citations are formatted as follows: "Data ref: Smith et al, 2001" or "Data ref: NCBI Sequence Read Archive PRJNA342805, 2017". In the Reference list, data citations must be labeled with "[DATASET]". A data reference must provide the database name, accession number/identifiers and a resolvable link to the landing page from which the data can be accessed at the end of the reference. Further instructions are available at <https://www.embopress.org/page/journal/14693178/authorguide#referencesformat>;.

Not applicable to our study.

10) As part of the EMBO publication's Transparent Editorial Process, EMBO reports publishes online a Review Process File to accompany accepted manuscripts. This File will be published in conjunction with your paper and will include the referee reports, your point-by-point response and all pertinent correspondence relating to the manuscript.

You are able to opt out of this by letting the editorial office know (emboreports@embo.org). If you do opt out, the Review Process File link will point to the following statement: "No Review Process File is available with this article, as the authors have chosen not to make the review process public in this case."

11) Finally, EMBO reports papers are accompanied online by A) a short (1-2 sentences) summary of the findings and their significance, B) 2-3 bullet points highlighting key results and C) a synopsis

image that is 550x200-600 pixels large (width x height) in .png format. You can either show a model or key data in the synopsis image. Please note that the size is rather small and that text needs to be readable at the final size. Please send us this information along with the revised manuscript.

We composed and uploaded a synopsis image together with a short summary.

Manuscript number: EMBOR-2021-53968V2
Title: Tet enzymes are essential for early embryogenesis and completion of embryonic genome activation
Author(s): Julia Arand, H. Rosaria Chiang, David Martin, Michael Snyder, Julien Sage, Renee Reijo Pera, and Mark Wossidlo

Dear Mark,

Thank you for your patience while we have editorially checked and reviewed your revised manuscript and point-by-point response. As you know, we perform a number of quality checks on manuscript figures and text, and there are some minor issues that still need your attention.

I am thus writing with an 'accept in principle' decision, which means that I will be happy to accept your manuscript for publication once a few minor issues/corrections have been addressed, as follows.

1) I attach to this email a related manuscript file with comments by our data editors. Please address all comments and upload a revised file with tracked changes with your final manuscript submission.

2) Please also address the following textual changes:

- References to preprints need to be clearly marked following this template:
• In-text citation: (preprint: NAME1 et al, YEAR)
• Author NAME1, Author NAME2, (YEAR) article title. bioRxiv doi: 1234/002.dfj123 [PREPRINT]

Thus, in the text Cotto et al needs to be cited (preprint: Cotto et al, 2021) and in the reference list you need to add the doi and the identifier [PREPRINT].

- Please add callouts to the panels of Appendix Fig S9.

- Please upload Tables Tables EV1, EV2, EV4, EV7, and EV8 as Dataset EV files. The nomenclature is Dataset EV# and the legend can stay as is, i.e., part of the Dataset .xls file.

- Please change the names of Appendix Figs S1 and S4 to Appendix table S1 and S2 and please also update the callouts in the text accordingly. I also noticed a type on the legend of Appendix Fig. S9 ("each dots represents one ..." should be "each dot represents....")

Once you have made these minor revisions, please use the following link to submit your corrected manuscript:

Link Not Available

If all remaining corrections have been attended to, you will then receive an official decision letter from the journal accepting your manuscript for publication in the next available issue of EMBO reports. This letter will also include details of the further steps you need to take for the prompt inclusion of your manuscript in our next available issue.

Thank you for your contribution to EMBO reports.

Kind regards,
Martina

Martina Rembold, PhD
Senior Editor
EMBO reports

The authors performed the requested editorial changes.

2nd Revision - Editorial Decision **18th Nov 2021**

Dr. Mark Wossidlo
Medical University of Vienna
Vienna A-1090
Austria

Dear Mark,

Thank you for implementing the final minor corrections. I am now very pleased to accept your manuscript for publication in the next available issue of EMBO reports. Thank you for your contribution to our journal.

At the end of this email I include important information about how to proceed. Please ensure that you take the time to read the information and complete and return the necessary forms to allow us to publish your manuscript as quickly as possible.

As part of the EMBO publication's Transparent Editorial Process, EMBO reports publishes online a Review Process File to accompany accepted manuscripts. As you are aware, this File will be published in conjunction with your paper and will include the referee reports, your point-by-point response and all pertinent correspondence relating to the manuscript.

If you do NOT want this File to be published, please inform the editorial office within 2 days, if you have not done so already, otherwise the File will be published by default [contact: emboreports@embo.org]. If you do opt out, the Review Process File link will point to the following statement: "No Review Process File is available with this article, as the authors have chosen not to make the review process public in this case."

Should you be planning a Press Release on your article, please get in contact with emboreports@wiley.com as early as possible, in order to coordinate publication and release dates.

Thank you again for your contribution to EMBO reports and congratulations on a successful publication. Please consider us again in the future for your most exciting work.

Kind regards,

Martina

Martina Rembold, PhD
Senior Editor
EMBO reports

****

THINGS TO DO NOW:

You will receive proofs by e-mail approximately 2-3 weeks after all relevant files have been sent to our Production Office; you should return your corrections within 2 days of receiving the proofs.

Please inform us if there is likely to be any difficulty in reaching you at the above address at that time. Failure to meet our deadlines may result in a delay of publication, or publication without your corrections.

All further communications concerning your paper should quote reference number EMBOR-2021-53968V3 and be addressed to emboreports@wiley.com.

Should you be planning a Press Release on your article, please get in contact with emboreports@wiley.com as early as possible, in order to coordinate publication and release dates.

EMBO PRESS

YOU MUST COMPLETE ALL CELLS WITH A PINK BACKGROUND

PLEASE NOTE THAT THIS CHECKLIST WILL BE PUBLISHED ALONGSIDE YOUR PAPER

Corresponding Author Name: Mark Wossidlo
Journal Submitted to: EMBO reports
Manuscript Number: EMBOR-2021-53968V1

Reporting Checklist For Life Sciences Articles (Rev. June 2017)

This checklist is used to ensure good reporting standards and to improve the reproducibility of published results. These guidelines are consistent with the Principles and Guidelines for Reporting Preclinical Research issued by the NIH in 2014. Please follow the journal's authorship guidelines in preparing your manuscript.

A- Figures
1. Data
The data shown in figures should satisfy the following conditions:

- the data were obtained and processed according to the field's best practice and are presented to reflect the results of the experiments in an accurate and unbiased manner.
- figure panels include only data points, measurements or observations that can be compared to each other in a scientifically meaningful way.
- graphs include clearly labeled error bars for independent experiments and sample sizes. Unless justified, error bars should not be shown for technical replicates.
- if n< 5, the individual data points from each experiment should be plotted and any statistical test employed should be justified
- Source Data should be included to report the data underlying graphs. Please follow the guidelines set out in the author ship guidelines on Data Presentation.

2. Captions

Each figure caption should contain the following information, for each panel where they are relevant:

- a specification of the experimental system investigated (eg cell line, species name).
- the assay(s) and method(s) used to carry out the reported observations and measurements
- an explicit mention of the biological and chemical entity(ies) that are being measured.
- an explicit mention of the biological and chemical entity(ies) that are altered/varied/perturbed in a controlled manner.

- the exact sample size (n) for each experimental group/condition, given as a number, not a range;
- a description of the sample collection allowing the reader to understand whether the samples represent technical or biological replicates (including how many animals, litters, cultures, etc.).
- a statement of how many times the experiment shown was independently replicated in the laboratory.
- definitions of statistical methods and measures:
  - common tests, such as t-test (please specify whether paired vs. unpaired), simple $\chi 2$ tests, Wilcoxon and Mann-Whitney tests, can be unambiguously identified by name only, but more complex techniques should be described in the methods section;
  - are tests one-sided or two-sided?
  - are there adjustments for multiple comparisons?
  - exact statistical test results, e.g., P values = x but not P values < x;
  - definition of 'center values' as median or average;
  - definition of error bars as s.d. or s.e.m.

Any descriptions too long for the figure legend should be included in the methods section and/or with the source data.

In the pink boxes below, please ensure that the answers to the following questions are reported in the manuscript itself. Every question should be answered. If the question is not relevant to your research, please write NA (non applicable). We encourage you to include a specific subsection in the methods section for statistics, reagents, animal models and human subjects.

USEFUL LINKS FOR COMPLETING THIS FORM

http://www.antibodypedia.com
http://1degreebio.org
http://www.equator-network.org/reporting-guidelines/improving-bioscience-research-repor
http://grants.nih.gov/grants/olaw/olaw.htm
http://www.mrc.ac.uk/Ourresearch/Ethicsresearchguidance/Useofanimals/index.htm
http://ClinicalTrials.gov
http://www.consort-statement.org
http://www.consort-statement.org/checklists/view/32-consort/66-title
http://www.equator-network.org/reporting-guidelines/reporting-recommendations-for-tumo
http://datadryad.org
http://figshare.com
http://www.ncbi.nlm.nih.gov/gap
http://www.ebi.ac.uk/ega
http://biomodels.net/
http://biomodels.net/miriam/
http://jjj.biochem.sun.ac.za
https://osp.od.nih.gov/biosafety-biosecurity-and-emerging-biotechnology/
http://www.selectagents.gov/

B- Statistics and general methods

Please fill out these boxes (Do not worry if you cannot see all your text once you press return)

1.a. How was the sample size chosen to ensure adequate power to detect a pre-specified effect size?	Sample size was chosen analogous to sample sizes used in general in the field and based on our experience.
1.b. For animal studies, include a statement about sample size estimate even if no statistical methods were used.	Sample size was chosen analogous to sample sizes used in general in the field and based on our experience.
2. Describe inclusion/exclusion criteria if samples or animals were excluded from the analysis. Were the criteria pre-established?	NA
3. Were any steps taken to minimize the effects of subjective bias when allocating animals/samples to treatment (e.g. randomization procedure)? If yes, please describe.	Oocytes from several female mice per experiment were pooled and randomly picked for each condition.
For animal studies, include a statement about randomization even if no randomization was used.	Oocytes from several female mice per experiment were pooled and randomly picked for each condition.
4.a. Were any steps taken to minimize the effects of subjective bias during group allocation or/and when assessing results (e.g. blinding of the investigator)? If yes please describe.	Blinding was not applied in the experiments
4.b. For animal studies, include a statement about blinding even if no blinding was done	Blinding was not applied in the experiments
5. For every figure, are statistical tests justified as appropriate?	Yes
Do the data meet the assumptions of the tests (e.g., normal distribution)? Describe any methods used to assess it.	Normal distribution was check with statistical test (Shapiro-Wilk) and/or visually checked with appropiate plots (Box-Plot/QQ-plot/Frequency distribution) for data sets analyzed with ANOVA or paired t-tests.
Is there an estimate of variation within each group of data?	Standard deviations are indicated in all analyzed datasets.

Is the variance similar between the groups that are being statistically compared?	Not always - test was choosen appropiately

C- Reagents

6. To show that antibodies were profiled for use in the system under study (assay and species), provide a citation, catalog number and/or clone number, supplementary information or reference to an antibody validation profile. e.g., Antibodypedia (see link list at top right), 1DegreeBio (see link list at top right).	We provide this information in the method section.
7. Identify the source of cell lines and report if they were recently authenticated (e.g., by STR profiling) and tested for mycoplasma contamination.	NA

* for all hyperlinks, please see the table at the top right of the document

D- Animal Models

8. Report species, strain, gender, age of animals and genetic modification status where applicable. Please detail housing and husbandry conditions and the source of animals.	We provide this information in the method section.
9. For experiments involving live vertebrates, include a statement of compliance with ethical regulations and identify the committee(s) approving the experiments.	All mouse experiments were carried out in accordance to the Stanford University Administrative Panel on Laboratory Animal Care and the Medical University in agreement with the authorizing committees.
10. We recommend consulting the ARRIVE guidelines (see link list at top right) (PLoS Biol. 8(6), e1000412, 2010) to ensure that other relevant aspects of animal studies are adequately reported. See author guidelines, under 'Reporting Guidelines'. See also: NIH (see link list at top right) and MRC (see link list at top right) recommendations. Please confirm compliance.	We follow the ARRIVE guidelines.

E- Human Subjects

11. Identify the committee(s) approving the study protocol.	NA
12. Include a statement confirming that informed consent was obtained from all subjects and that the experiments conformed to the principles set out in the WMA Declaration of Helsinki and the Department of Health and Human Services Belmont Report.	NA
13. For publication of patient photos, include a statement confirming that consent to publish was obtained.	NA
14. Report any restrictions on the availability (and/or on the use) of human data or samples.	NA
15. Report the clinical trial registration number (at ClinicalTrials.gov or equivalent), where applicable.	NA
16. For phase II and III randomized controlled trials, please refer to the CONSORT flow diagram (see link list at top right) and submit the CONSORT checklist (see link list at top right) with your submission. See author guidelines, under 'Reporting Guidelines'. Please confirm you have submitted this list.	NA
17. For tumor marker prognostic studies, we recommend that you follow the REMARK reporting guidelines (see link list at top right). See author guidelines, under 'Reporting Guidelines'. Please confirm you have followed these guidelines.	NA

F- Data Accessibility

18: Provide a "Data Availability" section at the end of the Materials & Methods, listing the accession codes for data generated in this study and deposited in a public database (e.g. RNA-Seq data: Gene Expression Omnibus GSE39462, Proteomics data: PRIDE PXD000208 etc.) Please refer to our author guidelines for 'Data Deposition'.<br><br>Data deposition in a public repository is mandatory for:<br>a. Protein, DNA and RNA sequences<br>b. Macromolecular structures<br>c. Crystallographic data for small molecules<br>d. Functional genomics data<br>e. Proteomics and molecular interactions	Data availability section is included in the manuscript: The datasets produced in this study are available in the following databases:<br>RNA-seq data: Gene Expression Omnibus GSE57063<br>(https://www.ncbi.nlm.nih.gov/geo/query/acc.cgi?acc=GSE57063)<br>Whole genome bisulfite-sequencing: Gene Expression Omnibus GSE156006<br>(https://www.ncbi.nlm.nih.gov/geo/query/acc.cgi?acc=GSE156006)
19. Deposition is strongly recommended for any datasets that are central and integral to the study; please consider the journal's data policy. If no structured public repository exists for a given data type, we encourage the provision of datasets in the manuscript as a Supplementary Document (see author guidelines under 'Expanded View' or in unstructured repositories such as Dryad (see link list at top right) or Figshare (see link list at top right).	Datasets were deposited to Gene Expression Omnibus and selected critical analyses are included in EV Tables. We furthermore provide EV Movies.
20. Access to human clinical and genomic datasets should be provided with as few restrictions as possible while respecting ethical obligations to the patients and relevant medical and legal issues. If practically possible and compatible with the individual consent agreement used in the study, such data should be deposited in one of the major public access-controlled repositories such as dbGAP (see link list at top right) or EGA (see link list at top right).	NA
21. Computational models that are central and integral to a study should be shared without restrictions and provided in a machine-readable form. The relevant accession numbers or links should be provided. When possible, standardized format (SBML, CellML) should be used instead of scripts (e.g. MATLAB). Authors are strongly encouraged to follow the MIRIAM guidelines (see link list at top right) and deposit their model in a public database such as Biomodels (see link list at top right) or JWS Online (see link list at top right). If computer source code is provided with the paper, it should be deposited in a public repository or included in supplementary information.	NA

G- Dual use research of concern

22. Could your study fall under dual use research restrictions? Please check biosecurity documents (see link list at top right) and list of select agents and toxins (APHIS/CDC) (see link list at top right). According to our biosecurity guidelines, provide a statement only if it could.	NA
